# Influence of different lactic acid bacteria strains and milling process on the solid-state fermented green and red lentils (*Lens culinaris* L.) properties including gamma-aminobutyric acid formation

**DOI:** 10.3389/fnut.2023.1118710

**Published:** 2023-04-13

**Authors:** Ernestas Mockus, Egle Zokaityte, Vytaute Starkute, Dovile Klupsaite, Romas Ruibys, João Miguel Rocha, Vadims Bartkevics, Elena Bartkiene

**Affiliations:** ^1^Institute of Animal Rearing Technologies, Faculty of Animal Sciences, Lithuanian University of Health Sciences, Kaunas, Lithuania; ^2^Department of Food Safety and Quality, Veterinary Academy, Lithuanian University of Health Sciences, Kaunas, Lithuania; ^3^Institute of Agricultural and Food Sciences, Agriculture Academy, Vytautas Magnus University, Kaunas, Lithuania; ^4^Universidade Católica Portuguesa, CBQF - Centro de Biotecnologia e Química Fina – Laboratório Associado, Escola Superior de Biotecnologia, Rua Diogo Botelho, Porto, Portugal; ^5^Animal Health and Environment “BIOR”, Institute of Food Safety, Riga, Latvia

**Keywords:** lentils (*Lens culinaris* L.), solid-state fermentation, lactic acid bacteria, gamma-aminobutyric acid, biogenic amines

## Abstract

The aim of this study was to evaluate the influence of lactic acid bacteria (LAB) strains (*Lactiplantibacillus plantarum* No.122 and *Lacticaseibacillus casei* No.210) and milling process on the solid-state fermented (for 24 h, at 30°C) green and red lentils (*Lens culinaris* L.) properties, chiefly pH, LAB viable counts, color coordinates, free amino acid (FAA) profile, γ-aminobutyric acid (GABA) and biogenic amine (BA) concentrations, fatty acid (FA) and volatile compound (VC) profiles. Results showed that both of the tested LAB strains are suitable for the fermentation of lentils: pH of fermented lentils was <4.5 and LAB viable counts >8.0 log_10_ colony-forming units (CFU)/g. A very strong negative correlation was found (*r* = −0.973, *p* ≤ 0.0001) between LAB counts and pH of the samples. Also, fermentation and milling process were significant factors toward color coordinates of the lentils. In most of the cases, solid-state fermentation (SSF) increased essential FAA content in lentils; however, some of the non-essential FAA content was reduced. SSF significantly increased GABA concentration in lentils and milling process was a significant factor on GABA content of the samples (*p* ≤ 0.05). The main BA in lentils was spermidine, and SSF decreased their total BA content (34.8% on average in red lentils and 39.9% on average in green lentils). The main FA in lentils were linoleic and oleic. The main VC in lentils were hexanal, 1-hexanol, hexanoic acid, D-limonene and (E)-2-nonen-1-ol. Furthermore, most of the VC showed significant correlations with pH of lentil samples, LAB counts and FA content. Finally, the LAB strain used for fermentation and the milling process of lentils are significant factors for most of the analyzed parameters in lentil. Moreover, despite the higher GABA concentration found in green non-milled SSF lentils, application of combined milling and SSF is recommended because they showed the lowest BA content in addition to higher essential FAA and GABA concentrations.

## 1. Introduction

Lentils (*Lens culinaris* L.; Family: *Fabaceae*) are used as a very valuable stock for human (1) and animal nutrition (2). Nowadays, these plant species are diversified (3) and well known for their rich dietary compositions (4). Depending on the cultivar., lentils can be yellow, orange, red, green, brown or black (5). According to the Food and Agriculture Organization (FAO), the global production of the lentils is primarily cultivated and harvested in Canada and India, which are estimated to be 1.99 and 1.1 million metric tons, respectively (6). There has been growing scientific interest to study lentils as a functional material due to their high biological value, as well as the presence of bioactive compounds (1).

Lentils have low fat content and their fatty acid (FA) fraction comprising 16.7% of saturated, 23.7% of monounsaturated and 58.8% of polyunsaturated FA (7). However, the composition of lentils varies widely, *viz.*: protein from 15.9 to 31.4%, carbohydrates from 43.4 to 74.9%, fat from 0.3 to 3.5%, total fiber content from 5.1 to 26.6% and ash content from 2.2 to 6.4% (8). These variations are explained by plant genetics, agri-ecological factors and production practices, as well as biotic and abiotic stresses (8). According to the nutrient data of US Department of Agriculture, raw lentil contains 24.6% protein, 63.4% carbohydrates, 1.1% fat and 2.7% ash (9). Despite that, in comparison with wheat or rice, lentils are richer in protein and lower in carbohydrates (10).

To increase the functional value, lentils can be fermented. Indeed, fermentation is an effective technology to lead many beneficial characteristics of the fermentable substrate, including higher quantities of phenolic compounds (11, 12) and better antioxidant properties (13, 14). In comparison submerged and solid-state fermentation (SSF), the latter is more sustainable because of the lower water content, smaller fermentation vessels used, in addition to the fact that during SSF various enzymes are excreted in higher concentrations by the existing microorganisms (15). Also, during the fermentation, antinutritional compounds are significantly degraded (11, 16).

Though many studies on modeling, the nutritional and functional characteristics of lentils *via* SSF have been carried out, to the best of our knowledge, changes in free amino acid (FAA) profile, γ-aminobutyric acid (GABA) and biogenic amine (BA) concentrations, fatty acid (FA) and volatile compound (VC) profiles due to SSF with *Lactiplantibacillus plantarum* No. 122 and *Lacticaseibacillus casei* No. 210 and milling process of the fermentable substrate have not been discussed so far. Also, to evaluate an influence of milling process is very important. Particle size can be of significance when biological treatment is applied, because of microorganism nutrients accessibility, technological starters can show different excretion properties of the enzymes, as well as other metabolites, which can lead to different properties of the fermented substrate.

The most abundant amino acid (AA) in lentils is glutamic acid, followed by aspartic acid, arginine, leucine and lysine. However, methionine and tryptophan are limiting AA in lentils (10). Taking into consideration the AA profile of lentils, we hypothesized in this study that SSF of lentils can be a good mean to increase the content of FAA as well as GABA. GABA is a potent bioactive compound which is most commonly produced *via* decarboxylation of glutamate, and lactic acid bacteria (LAB) strains are most widely used for producing GABA-enriched products by fermentation (17). Despite, that most of the production studies on GABA synthesis have been reported by submerged fermentation, SSF is more suitable in cost effective GABA production (10). It was reported that *Limosilactobacillus fermentum*, *Lactobacillus delbrueckii* subsp. *lactis*, *Lactococcus lactis*, *Pediococcus pentosaceus*, *Limosilactobacillus reuteri*, *Bifidobacterium* spp., *Levilactobacillus brevis*, *Pediococcus acidilactici* and *Latilactobacillus sakei* are the most popular LAB for GABA production (18, 19). Despite LAB fermentation technology has a Generally Regarded As Safe (GRAS) status, there are two main metabolic pathways—chiefly glutamate decarboxylase pathway and putrescine pathway—which are followed by microorganisms in the production of GABA, and during the same pathways BA can be formed (20). In the case of fermented substrates, decarboxylase-producing microorganisms can be used as technological starter cultures (21). From here, on may conclude that the control of BA concentration in the end-product is required.

Under this context, the present research study was carried out to evaluate the influence of two LAB strains (*Lactiplantibacillus plantarum* No.122 and *Lacticaseibacillus casei* No.210) and milling process on the properties (pH, LAB count, color coordinates, FAA profile, GABA and BA concentrations, FA and VC profiles) of green and red lentils (*Lens culinaris* L.) subjected to SSF.

## 2. Materials and methods

### 2.1. Characteristics of the lentils, lactic acid bacteria used for fermentation of the lentils and fermentation conditions

Green (variety ‘CDC Lemay’) and red (variety ‘CDC Red Rider’) lentils (composition per 100 g of the green lentils: total carbohydrates 48.5 g, protein 24.0 g, fat 1.5 g; composition per 100 g of the red lentils: total carbohydrates 25.0 g, protein 13.0 g, fat 0.7 g) were provided by Ltd. ‘Galinta ir partneriai’ (Kaunas, Lithuania). To evaluate the influence of the milling process, samples were grounded with a Laboratory Mill 120 (Perten Instruments AB, Stockholm, Sweden) to 1–2 mm particle size.

The LAB strains (*Lactiplantibacillus plantarum* No. 122 and *Lacticaseibacillus casei* No. 210) were acquired from the Lithuanian University of Health Sciences collection (Kaunas, Lithuania). Before the experiment, LAB strains were incubated and multiplied in De Man, Rogosa and Sharpe (MRS) broth culture medium (Biolife, Milano, Italy) at 30°C under anaerobic conditions for 24 h. A total of 3 mL of fresh viable LAB grown on MRS broth (average cell concentration of 8.6 log_10_ CFU mL) were inoculated in 100 g of lentils (lentils/water ratio was 1:1, w/w), where the final densities of the viable LAB strain in the lentils-water mixtures were on average 5.02 (red lentils) and 4.98 (green lentils) log_10_ CFU/g. Afterwards, the lentil samples were fermented under anaerobic conditions in a chamber incubator without agitation (Memmert GmbH Co. KG, Schwabach, Germany) for 24 h, at 30°C. Non-fermented lentil samples (mixed with water) were analyzed as the control.

The applied experimental design gave rise to a total of 10 samples, chiefly: non-treated (i.e. non-milled and non-fermented) red and green lentils (Re and Gr, respectively); non-milled red and green lentils (solid-state) fermented with No.122 and No. 210 LAB strains (Re_122_, Re_210_, Gr_122_, Gr_210_, respectively); milled red and green lentils (solid-state) fermented with No.122 and No. 210 LAB strains (Re_122milled_, Re_210milled_, Gr_122milled_, Gr_210milled_, respectively).

**Figure 1:**
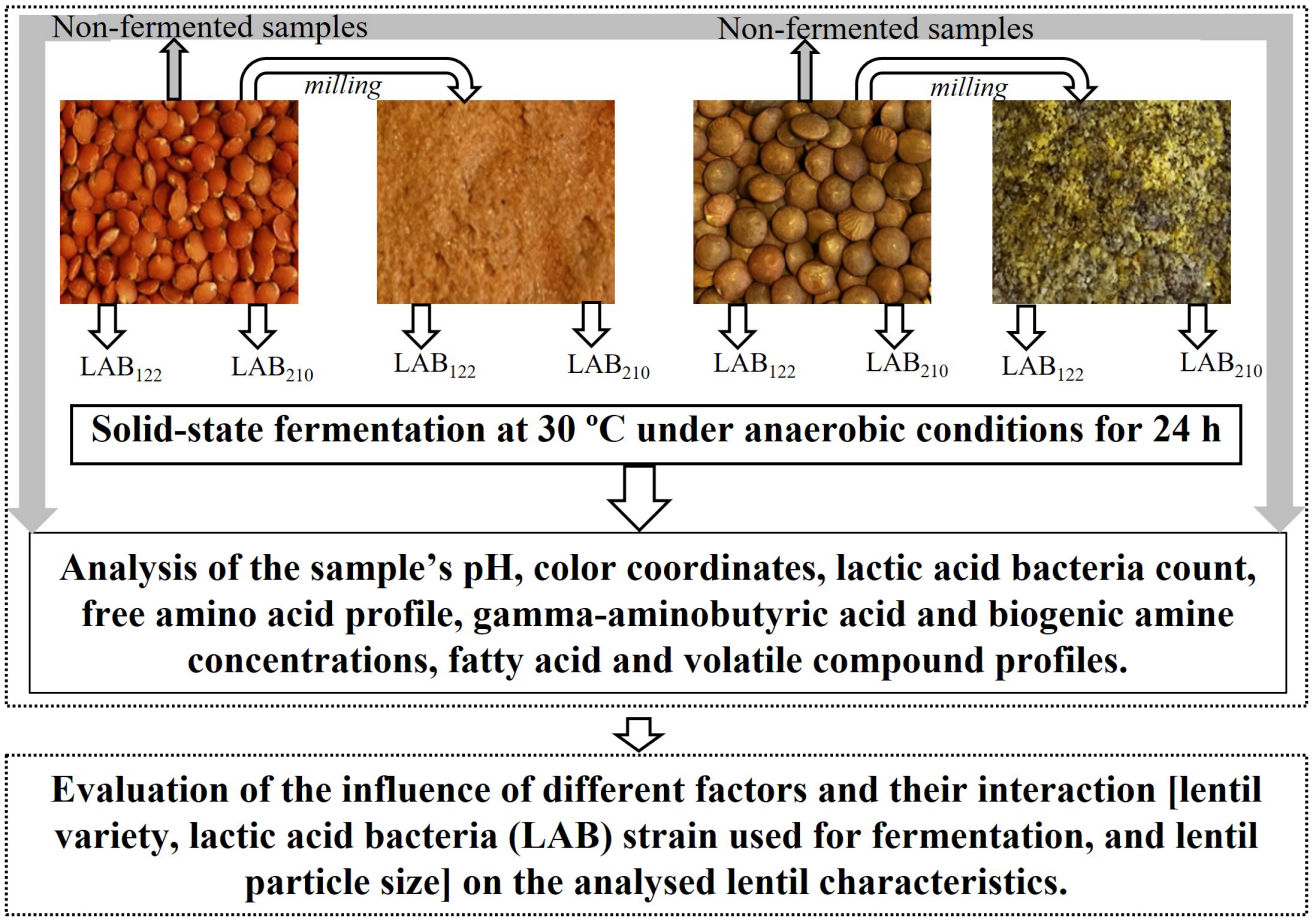
Experimental design (LAB—lactic acid bacteria strain; _122_—*Lactiplantibacillus plantarum* strain; _210_—*Lacticaseibacillus casei* strain).

Before and after fermentation, the pH, color coordinates, LAB viable counts, FAA profile, GABA and BA concentrations, FA and VC profiles of the lentil samples were analyzed. The experimental design is schematised in Figure 1.

### 2.2. Analysis of pH, color coordinates, and lactic acid bacteria viable counts in the lentil samples

The pH of lentil samples was evaluated using a pH meter (Inolab 3, Hanna Instruments, Venet, Italy) by inserting the pH electrode into the lentil samples. The color coordinates of the lentil samples were evaluated on the surface using the CIE L*a*b* system (CromaMeter CR-400, Konica Minolta, Marunouchi, Tokyo Japan). The LAB viable counts were determined according to the method described by Bartkiene et al. (22).

Evaluation of free amino acid (FAA) profile and gamma-aminobutyric acid (GABA) concentration in the lentil samples.

Sample preparation and dansylation was performed according to the method of Ben-Gigirey et al. (23) with some modifications here described. Homogenizsd sample (~ 1,000 mg) was weighted in a 15 mL sample tube and analytes were extracted with 10 mL of aqueous 0.1 M HCl solution by shaking for 1 h. Resultant mixture was centrifuged at 4,000 rpm for 10 min. For derivatization, 100 μL of resultant supernatant was diluted to 500 μL with 0.1 M HCl solution. Resultant mixture was alkalinised by adding 40 μL of 2 M NaOH and 70 μL of saturated NaHCO_3_ solution. Derivatization was performed by adding 1 mL of 10 mg/mL dansyl chloride solution in acetonitrile and heating the resulting mixture at 60°C for 30 min. Reaction mixture was quenched using 50 μl of 25% ammonia solution and filtered through a 0.22 μm membrane filter to the autosampler vial. Concentration of analytes were determined using a Varian ProStar HPLC system (Varian Corp., Palo Alto, California, USA, two ProStar 210 pumps, a ProStar 410 autosampler) and Thermo Scientific LCQ Fleet Ion trap mass detector. For analyte detection, the mass spectrometer operated at positive ionisation single ion monitoring mode and single reaction monitoring mode (for glutamine). Concentration of analytes was determined using the standard addition method by spiking extract with known concentration of analytes. For the separation of derivatives, a Discovery^®^ HS C18 column (150 × 4.6 mm, 5 μm; SupelcoTM Analytical, Bellefonte, Pennsylvania, USA) was used. The mobile phase A was 0.1% formic acid in 5% aqueus acetonitrile and the mobile phase B was 0.1% acetonitrile. A flow-rate of 0.3 mL/min and an injection volume of 10 μL were used for analysis. The analytical gradient of the mobile phase was as follows: 0 to 10 min (linear gradient) 15 to 60% B, 10 to 40 min (linear gradient) 60 to 95% B and 40 to 48 min 95% B, followed by reequilibration for 10 min with 15% B (increased to 0.6 mL/min flow-rate). The limit of quantification (according to lowest concentration of constructed calibration curve) was 0.02 μmol/g.

### 2.3. Analysis of biogenic amine concentration in the lentil samples

Sample preparation and identification and quantification of the BA—which included tryptamine (TRP), phenylethylamine (PHE), putrescine (PUTR), cadaverine (CAD), histamine (HIS), tyramine (TYR), spermidine (SPRMD) and spermine (SPRM)—in lentil samples was conducted by following the experimental procedure reported by Ben-Gigirey et al. (24) with some modifications. Briefly, the standard BA solutions were prepared by dissolving known amounts of each BA (including internal standard) in 20 mL of deionised water. The extraction of BA in samples (5 g) was undertaken by using 0.4 mol/l perchloric acid. The derivatization of sample extracts and standards was performed using dansyl chloride solution (10 mg/mL) as reagent. The chromatographic analyses were carried out using a Varian ProStar HPLC system (Varian Corp., Palo Alto, California, USA) with two ProStar 210 pumps, a ProStar 410 auto-sampler, a ProStar 325 UV/VIS Detector and Galaxy software (Agilent, Santa Clara, California, USA) for data processing. For the separation of amines, a Discovery^®^ HS C18 column (150 × 4.6 mm, 5 μm; SupelcoTM Analytical, Bellefonte, Pennsylvania, USA) was used. The eluents of the mobile phase were ammonium acetate (A) and acetonitrile (B) and the elution programme consisted of a gradient system at 0.8 mL/min flow-rate. The detection wavelength was set to 254 nm, the oven temperature was 40°C and samples were injected in 20 μl aliquots. The target compounds were identified based on their retention times in comparison to their corresponding standards.

### 2.4. Analysis of fatty acid profile in the lentil samples

The extraction of lipids for FA quantification was undertaken with chloroform/methanol (2:1, v/v) and fatty acid methyl esters (FAME) were prepared according to the protocol described by Pérez-Palacios et al. (25). The FA composition of the lentil samples was identified using a gas chromatograph GC-2010 Plus (Shimadzu Europa GmbH, Duisburg, Germany) equipped with a mass spectrometer GCMS-QP2010 (Shimadzu Europa GmbH, Duisburg, Germany). Separation was carried out on a Stabilwax-MS column (30 m length, 0.25 mmID and 0.25 μm df) (Restek Corporation, Bellefonte, US). The mass spectrometer operated at full scan mode and the analyte was injected in split mode at 1:60 split ratio. The following parameters were used: MS ion source temperature: 240°C; MS interface temperature 240°C; helium (carrier gas) flow-rate: 0.90 mL/min; injector: 240°C and oven temperature programme was: 50°C (4 min), 10°C/min to 110°C (1 min), 15°C/min to 160°C (2 min), 2.5°C/min to 195°C (1 min), 2°C/min to 230°C (1 min) and 2°C/min to 240°C (12 min). The individual FAME peaks were identified by comparing their retention times with FAME standards (Merck & Co., Inc., Kenilworth, NJ, USA). The quantification was determined by using the corrected area normalization method.

### 2.5. Analysis of volatile compound profile in the lentil samples

The VC of the lentil samples were analyzed by gas chromatography–mass spectrometry (GC–MS). A solid-phase microextraction (SPME) device with Stableflex™ fiber coated with a 50 μm PDMS-DVB-Carboxen™ layer (Supelco, USA) was used for analysis. For headspace extraction, 1 g of sample and 10 mL of 1 M phosphate buffer (pH = 3) were transferred to the 20 mL extraction vial, mixed, sealed with a polytetrafluoroethylene septum and thermostated at 60°C for 30 min before exposing the fiber in the headspace. The fiber was exposed to the headspace of the vial for 10 min and desorbed in an injector liner for 2 min (splitless injection mode). Prepared samples were analyzed with a GCMS-QP2010 (Shimadzu, Japan) gas chromatograph coupled with a mass spectrometer. The following conditions were used for analysis: injector temperature 250°C; ion source temperature 220°C and interface temperature 260°C. Helium was used as the carrier gas at 0.65 mL/min flowrate. For separation of VC, a low polarity Rxi^®^-5MS column (Restek, USA) (length 30 m, coating thickness 0.25 μm, inner diameter of 0.25 mm) was used. The temperature gradient was programmed from starting at 40°C (3 min hold) to 220°C (5°C/min) up to 310°C (15°/min) (6 min hold). The VC were identified according to mass spectrum libraries (NIST11, NIST11S, and FFNSC2). For identification purposes, alkane mix (C8-C20) was analyzed to obtain the retention indexes of unknown compounds.

### 2.6. Statistical analysis

Statistical analysis was completed using IBM SPSS Statistics for Windows, v28.0.1.0 (142) (SPSS, Chicago, Illinois, USA). The results were expressed as the mean values (for lentil samples *n* = 6; fermentation was performed two times, and from one substrate 3 samples were taken for analysis) ± standard error (SE). In order to evaluate the effects of different lentil cultivars, different LAB used for fermentation and milling process on the lentil quality parameters, data were analyzed by multivariate analysis of variance and Tukey-HSD tests as post-hoc tests. A linear Pearson’s correlation was used to quantify the strength of the relationship between the variables. The results were recognized as statistically significant at *p* ≤ 0.05.

## 3. Results and discussion

pH, color coordinates and lactic acid bacteria (LAB) viable counts in the lentil samples.

The pH, color coordinates, LAB viable counts and images of the lentil samples are given in Table 1. When comparing the red lentil samples, the lowest pH values were reached with Re_122_ samples (4.21), and samples Re_210_ and Re_122milled_ pH was, on average, 4.32. Regarding the green lentil group, the lowest pH values was attained in Gr_122milled_ samples (4.23), whereas in the other samples, the pH was 2.8, 3.3 and 4.0% higher (Gr_210milled_, Gr_122_ and Gr_210_, respectively). Most of the analyzed factors and their interactions were significant on the pH of the milling process (Supplementary Table S1.1).

**Table 1:** pH, color coordinates, lactic acid bacteria (LAB) viable counts and images of non-treated and fermented lentils.

Lentil samples	pH after 0 h	pH after 24 h	Color coordinates, NBS			LAB viable counts, log_10_ CFU/g
			L*	a*	b*	
	Parameters of the red lentil samples					
Re	6.14 ± 0.02^e^	-	55.90 ± 2.34^b^	24.04 ± 0.98^g^	26.40 ± 1.03^f^	4.21 ± 0.33^a^
Re_122_	5.79 ± 0.01^a,b^	4.21 ± 0.02^a^	53.19 ± 1.98^b^	19.59 ± 1.32^e^	22.79 ± 0.87^e^	8.04 ± 0.27^b^
Re_210_	5.98 ± 0.02^d^	4.33 ± 0.01^b^	53.23 ± 1.45^b^	20.92 ± 1.96^f^	23.46 ± 0.67^e^	8.15 ± 0.19^b^
Re_122milled_	5.75 ± 0.03^a^	4.31 ± 0.03^b^	44.18 ± 1.54^a^	5.92 ± 0.47^c^	17.51 ± 0.37^c^	8.01 ± 0.34^b^
Re_210milled_	5.70 ± 0.02^a^	4.43 ± 0.02^c^	45.86 ± 2.01^a^	6.10 ± 0.52^c^	18.45 ± 0.38^d^	8.16 ± 0.29^b,c^
	Parameters of the green lentil samples					
Gr	6.43 ± 0.02^f^	-	42.88 ± 1.98^a^	6.28 ± 0.38^c^	14.32 ± 0.25^a^	4.10 ± 0.25^a^
Gr_122_	5.81 ± 0.03^b^	4.37 ± 0.02^b,c^	58.55 ± 2.05^b^	16.94 ± 1.30^d^	25.91 ± 0.58^f^	8.34 ± 0.28^b,c^
Gr_210_	5.88 ± 0.01^c^	4.40 ± 0.03^c^	57.98 ± 2.93^b^	15.93 ± 1.22^d^	25.83 ± 0.76^f^	8.37 ± 0.30^b,c^
Gr_122milled_	5.99 ± 0.01d	4.23 ± 0.01^a^	52.61 ± 3.27^b^	1.87 ± 0.11^b^	18.91 ± 0.64^d^	8.59 ± 0.26^b,c^
Gr_210milled_	5.82 ± 0.04b	4.35 ± 0.02^b^	52.68 ± 2.62^b^	1.40 ± 0.20^a^	15.57 ± 0.31^b^	8.60 ± 0.17^c^
Images of the non-treated and treated lentils						
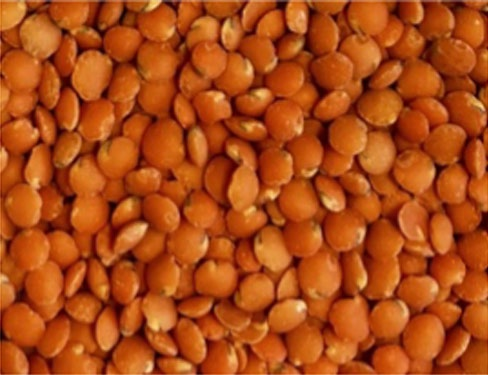	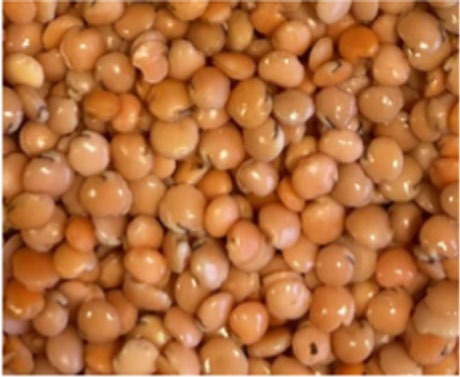		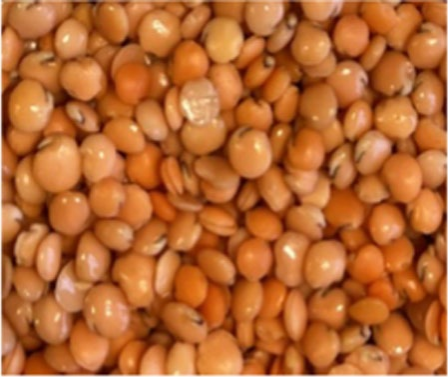	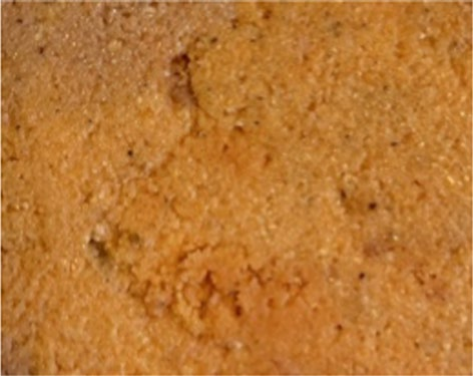	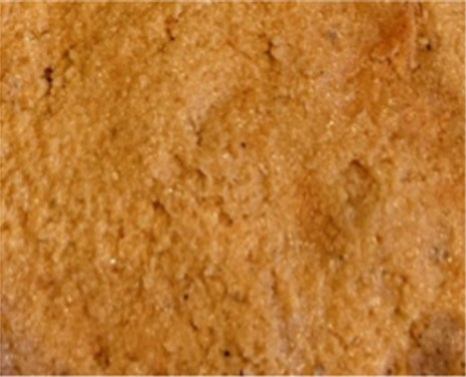	
Re	Re_122_		Re_210_	Re_122milled_	Re_210milled_	
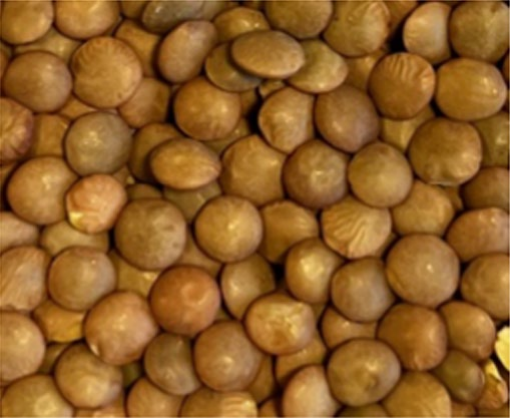	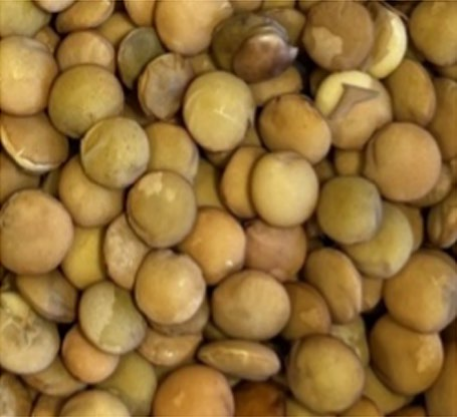		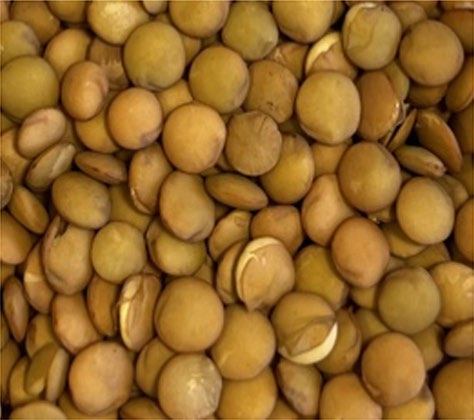	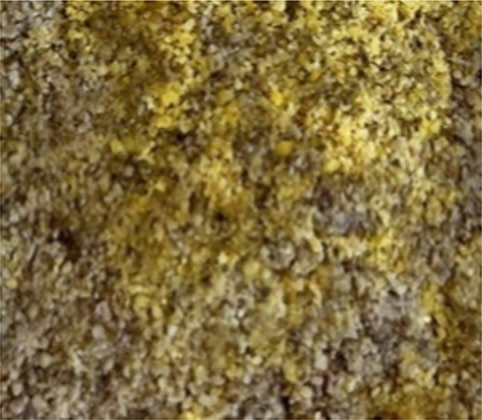	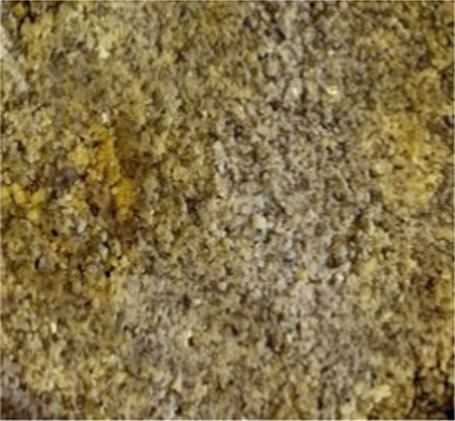	
Gr	Gr_122_		Gr_210_	Gr_122milled_	Gr_210milled_	

L* – lightness; a* – redness (−a* greenness); b* – yellowness (−b* blueness); LAB, lactic acid bacteria; NBS, National Bureau of Standards units; CFU, colony forming units; Re, non-treated red lentils; Gr, non-treated green lentils; _122_ – fermented with Lactiplantibacillus plantarum strain; _210_ – fermented with Lacticaseibacillus casei strain; _milled_ – milled lentils. Data are represented as means (*n* = 6) ± SE. -not analyzed; ^a–g^Means with different letters in the column are significantly different all sample groups (*p* ≤ 0.05).

In comparison LAB count in all the lentil groups, in all the SSF groups, LAB count was above 8.0 log_10_ CFU/g, and the milling process was not a significant factor on LAB count in lentils (Supplementary Table S1.1). However, between the LAB viable counts and pH, a very strong negative correlation was found (*r* = −0.973, *p* ≤ 0.0001).

Mousavi et al. reported that the beverages produced by 100% of lentil and fermented with *Bifidobacterium bifidum* displayed significantly higher acidity values, in comparison with beverages produced with lower content of lentil flours (26). Also, the number of lactobacilli and bifidobacteria (after 24 h of fermentation) in beverages was over 9.0 log_10_ CFU/mL. Another study reported that the *Lp. plantarum* TK9 and *Lacticaseibacillus paracasei* TK1501 strains are suitable to ferment lentils both under liquid-state and solid-state fermentation conditions and LAB viable counts were higher than 8.0 log_10_ CFU/g (27). Our study showed that despite the low pH values of the fermented samples, viable LAB counts over than 8.0 log_10_ CFU/g were established. This finding can be explained by the high tolerance to acidic conditions of the LAB strains used for fermentation. Our previous studies showed that *Lactiplantibacillus plantarum* 122 and *Lacticaseibacillus casei* 210 strains are versatile technological microorganisms with high-acidity resistance (28). Nevertheless, the adaptation of LAB to low pH conditions depends on the environmental factors and phenotype traits of the strains (29). This can explain different results reported in different studies. Indeed, LAB strain and milling process can be manipulated to obtain the most appropriated characteristics of the end products, including high viable counts of LAB.

Observing the lightness (L*) of red lentil samples, Re_122milled_ and Re_210milled_ showed, on average, 16.8% lower values than non-treated (Re) and non-milled (solid-state) fermented samples (Re_122_ and Re_210_). In all the cases, fermentation decreased red lentil redness (a*), and the lowest a* coordinates were attained in the milled and fermented samples (i.e., Re_122milled_ and Re_210milled_ samples with a* coordinates of 45.02 NBS, on average). Similar tendencies were found of the red lentil yellowness (b*), and the lowest b* coordinates were found in Re_122milled_ samples (17.51 NBS).

In what concerns to the green lentil group, in all cases the fermentation process increased L* and, in comparison with the non-treated green lentil sample, fermented samples showed, on average, 29.3% higher L* values. However, significant differences on L* coordinate between non-milled- and milled-fermented samples were not observed. The lowest a* coordinate was obtained with the sample Gr_210milled_ (1.40 NBS), and the lowest b* coordinate was displayed by the green lentil control (14.32 NBS). In comparison with the non-treated sample (Gr), b* coordinates of fermented samples were 8.73% (in Gr_210milled_ sample) to 80.6% (in Gr_122_ and Gr_210_ samples) higher.

The color of the product is a very important quality indicator and critical to consumer’s sensory acceptance. The main colored compounds in lentils are carotenoids and tocopherols, and lentils are a good source of both (30). It was reported data about carotenoid and tocopherol compositions in ten red and ten green lentils (31). The predominant tocopherol in lentils was γ-tocopherol (96–98% of the total tocopherol content), followed by δ- and α-tocopherols (31). Changes in the color of lentils during the fermentation can be explained by their reaction with organic acids as well as by enzymatic hydrolysis. It was reported that the acidic additive or LAB inoculation can affect the α-tocopherol and β-carotene content of the substrate (32); however, the results from different studies are inconsistent.

The microorganism strains used for fermentation can utilize phytochemicals and lead to their degradation (33, 34). Hubert et al. reported that a decrease in tocopherols can be obtained during the soybean germ lactofermentation (33). Our study showed that significant factors on L* of the lentil samples were the LAB strain used for fermentation and the milling process (Supplementary Table S1.1). Finally, all the analyzed factors showed individually to significantly influence the a* and b* coordinates of lentils, but not all of their interactions were statistically significant (Supplementary Table S1.1).

### 3.1. Free amino acid profile and gamma-aminobutyric acid concentration in the lentil samples

**Figure 2:**
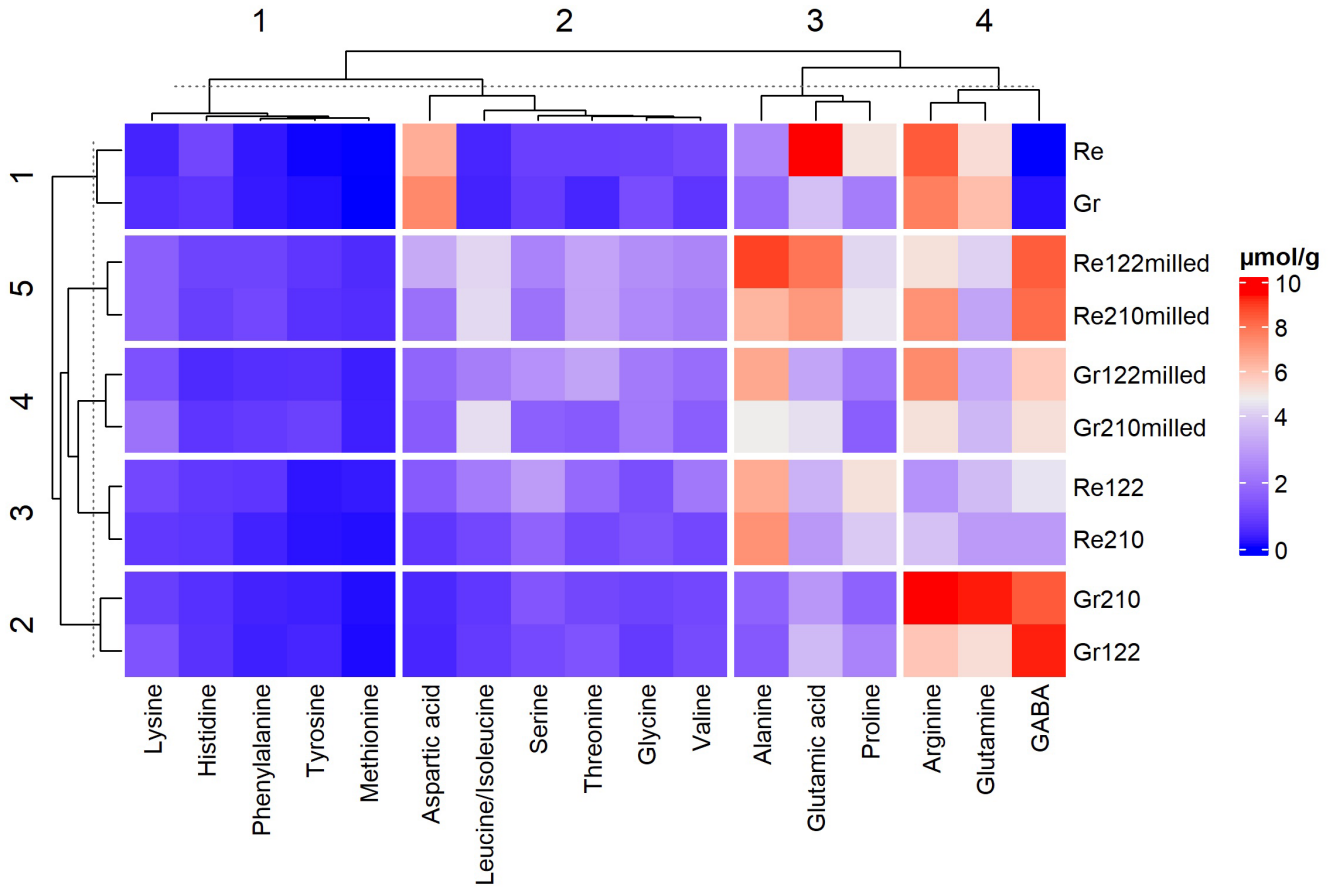
Gamma-aminobutyric acid (GABA) and free amino acid (FAA) content in lentil samples (Re—non-treated red lentils; Gr—non-treated green lentils; _122_—fermented with *Lactiplantibacillus plantarum* strain; _210_—fermented with *Lacticaseibacillus casei* strain; _milled_—milled lentils).

The content of GABA and FAA in lentil samples is given in Supplementary Table S2.1 and Figure 2.

In most of the cases, fermentation increased the essential FAA content in both type of lentils (red and green lentils), except valine in Re_210_ and histidine in Re_122_, Re_210_, Re_122milled_, and Re_210milled_ and Gr_122_, Gr_210_, Gr_122milled_ and Gr_210milled_ samples. In comparison with all the tested lentil groups, the highest content of threonine was found in Gr_122milled_ samples (3.13 μmol/g). The highest content of methionine and phenylalanine was found in red lentil samples Re_122milled_ and Re_210milled_ (on average, 0.560 and 1.04 μmol/g, respectively). In comparison with red and green lentil samples, in most of the cases (except Gr_210_ samples), higher concentration of valine was found in red lentil samples and fermentation increased valine content in samples by 2 times on average. The highest concentration of leucine/isoleucine was detected in Re_122milled_ and Re_210milled_, and Gr_210milled_ samples (on average, 4.32 μmol/g). However, the highest amount of lysine was displayed in Gr_210milled_ samples (2.02 μmol/g). Comparing histidine content in lentils, different tendencies were established: in 4 out of 10 analyzed sample groups, SSF decreased histidine content (in Re_122_, Re_210_, Re_210milled_, and Gr_122milled_ samples, on average, by 26.4, 32.0, 12.9, and 49.7%, respectively) in comparison with non-fermented ones.

Analyzing the content non-essential FAA in lentil samples, in all the cases, SSF increased serine and tyrosine content in samples in comparison with non-fermented ones. Comparing red lentil groups, SSF increased in every case glycine and alanine concentration (on average, from 1.4 to 2.7 times and from 2.6 to 3.7 times, respectively) and decreased glutamine and arginine (Arg) concentrations (on average, from 1.8 to 1.3 times and from 3.1 to 1.2 times, respectively), in comparison with the control. However, asparagine concentration was lower in both red and green lentils after SSF but Gr_210milled_ samples, in which asparagine content after SSF remained similar to the control group (on average, 4.10 μmol/g). In the majority of the SSF samples, glutamic acid concentration showed a tendency to reduce, except Gr_122_ and Gr_210_ – where, in these last samples, glutamic concentration remained similar (on average, 5.24 μmol/g) and slightly higher (by 31.1%), respectively, in comparison with control ones. Different trends were found in green lentil samples: glycine and alanine contents increased in both milled green lentil samples, however, proline (Pro) content decreased in both green lentil groups (non-milled and milled) fermented with No. 210 LAB strain. Opposite trends were observed in the arginine content in green lentil samples; moreover, arginine concentration increased after SSF with No. 210 strain but decreased after fermentation with No. 122 strain. Yet, in milled green lentil samples fermented with No. 210 strain, arginine concentration was found lower, in comparison with non-fermented ones.

As depicted in the Supplementary Table S1.2, most of the analyzed factors and their interactions were significant on the FAA concentration in lentil samples.

Looking at the GABA concentration in lentil samples, the values were always higher in SSF samples, when compared with non-fermented ones. In non-fermented red lentil samples, GABA was inexistent. However, in non-milled SSF samples with No. 122 and No. 210 strains, GABA content was, on average, 4.53 and 2.91 μmol/g, respectively. Milling process was proved to be a significant factor on GABA content of lentils (Supplementary Table S1.2). Indeed, in milled SSF red lentil samples higher GABA concentration was obtained (on average, by 1.87 and 2.79 times, in SSF with No. 122 and No. 210 strains, respectively). Furthermore, opposite tendencies were found in green lentil samples: non-milled SSF samples contained higher GABA concentration, in comparison with milled – non-fermented ones (by 1.62 and 1.63 times, in SSF with No. 122 and No. 210 strains samples, respectively).

Naturally, different fermentation designs lead to variations in the protein or amino acid profiles of fermentable substrate (35). Several studies reported that the protein concentration increases during fermentation (34, 36). However, antagonist results were also reported (36). These differences may be explained by the loss of dry matter, as a result of the microbial metabolic activities (35). Furthermore, the degradation of protein by microbial starters release FAA to the fermentable substrate (36). On the other hand, fermenting microorganisms can use FAA as a nutritional source (36, 37). Important to note that during fermentation the microorganisms increases the digestibility of plant proteins (36, 38). Also, the combination of fermentation with other processing technologies (for instance, thermal treatment) is more effective in reducing antinutritional factors (35). Pranoto et al. reported that *Lp*. *plantarum* can breakdown complex proteins, thereby releasing more peptides and FAA (36). As a matter of fact, during the fermentation a simultaneous increase and decrease of FAA in the fermentable substrate can be observed. Therefore, a control of the technological fermentation conditions as well as the quality parameters of the end-product are needed to avoid a loss of protein.

GABA production in fermented material also varied and these variations are related with many factors, including fermentation temperature, pH, substrate composition, process duration, *etc* (39). It was reported that the optimum process temperature for GABA synthesis is 30°C; however, other authors reported that the optimal temperature is 37°C (40). These differences can be related with the strains used for fermentation (28). The synthesis of GABA is catalyzed by the glutamic acid decarboxylase (18, 41, 42), which can be produced by LAB, yeasts and fungi (43–48). Though, decarboxylation of FAA can lead to BA formation. For this reason, this factor should be taken into consideration when selecting the most appropriate fermentation technology.

### 3.2. Biogenic amine concentration in the lentil samples

The BA concentrations in non-treated and fermented lentil samples are given in Table 2. Biogenic amines tryptamine (TRY), cadaverine (CAD), histamine (HIST) and tyramine (TYR) were not detected in lentil samples, and the main BA in lentil samples was spermidine (SPRMD). In all the cases SSF decreased SPRMD concentration in samples, and the lowest SPRMD content was found in Gr_122milled_ and Gr_210milled_ samples (on average, 108 mg/kg). Separate analyzed factors were significant on SPRMD concentration in lentil samples; conversely, their interactions were not significant on SPRMD formation (Supplementary Table S1.3). Between SPRMD concentration and pH of the samples, moderate positive correlation was found (*r* = 0.795, *p* ≤ 0.0001), as well as a strong negative correlation between SPRMD concentration and LAB count was established (*r* = −0.810, *p* ≤ 0.0001). In comparison spermine (SPRM) content in samples, all the SSF samples showed lower contents, in comparison with non-fermented ones. Furthermore, the lowest SPRM content was found in Gr_122milled_, Gr_210milled_ and Re_210milled_ samples (on average, 30.3 mg/kg). Likewise SPRMD, all the separate analyzed factors were significant on SPRM concentration in lentil samples; however, their interactions were not significant (Supplementary Table S1.3). Between the SPRM concentration and pH of the samples, a moderate positive correlation was found (*r* = 0.627, *p* ≤ 0.0001), as well as a strong negative correlation between SPRM concentration and LAB viable counts was recognized (*r* = −0.660, *p* ≤ 0.0001). Similar tendencies were established with putrescine (PUT). Particularly, SSF samples constantly showed lower PUT concentration, and the lowest content was found on both samples of red and green milled and fermented with both LAB strains (on average, 41.0 mg/kg). Significant influence on PUT concentration in lentils was obtained with the factors LAB strain used for fermentation and milling process (Supplementary Table S1.3). Also, strong positive correlation was found between the PUT concentration and samples pH (*r* = 0.844, *p* ≤ 0.0001), as well as a strong negative correlation between PUT concentration and LAB viable counts was attained (*r* = −0.829, *p* ≤ 0.0001). PHE concentration in red lentil samples ranged from 5.48 mg/kg (in Re_122_ and Re_210_ samples) to 11.3 mg/kg (in Re_210milled_ samples), whereas in green lentil samples ranged from 0.640 mg/kg (in Gr_210_ samples) to, on average, 9.09 mg/kg (in Gr and Gr_210milled_ samples). Finally, all the analyzed factors and their interactions were significant on PHE content in lentil samples (Supplementary Table S1.3).

**Table 2:** Biogenic amine (BA) concentration (mg/kg) in non-treated and fermented lentil samples.

Lentil samples	Biogenic amine, mg/kg							
	TRY	PHE	PUT	CAD	HIST	TYR	SPRMD	SPRM
	Parameters of the red lentil samples							
Re	nd	7.43 ± 0.65^e^	83.9 ± 5.3^d^	nd	nd	nd	235 ± 15.3^d^	69.1 ± 4.3^e^
Re_122_	nd	5.52 ± 0.32 ^c^	59.8 ± 3.8^b,c^	nd	nd	nd	168 ± 9.32^c^	55.0 ± 4.8^d^
Re_210_	nd	5.43 ± 0.41 ^c^	62.4 ± 4.5^c^	nd	nd	nd	172 ± 11.2^c^	55.8 ± 3.9^d^
Re_122milled_	nd	9.27 ± 0.71^f^	41.0 ± 2.9^a^	nd	nd	nd	142 ± 12.5^b^	36.1 ± 2.9^b^
Re_210milled_	nd	11.3 ± 0.11^g^	42.0 ± 3.1^a^	nd	nd	nd	134 ± 11.8^b^	33.5 ± 3.2^a,b^
	Parameters of the green lentil samples							
Gr	nd	9.49 ± 0.76^f^	82.8 ± 6.8^d^	nd	nd	nd	204 ± 16.3^d^	55.2 ± 4.6^d^
Gr_122_	nd	6.57 ± 0.54 ^d^	52.9 ± 3.9^b^	nd	nd	nd	138 ± 10.5^b^	42.7 ± 2.7^c^
Gr_210_	nd	0.640 ± 0.025^a^	53.8 ± 4.1^b^	nd	nd	nd	139 ± 11.4^b^	43.6 ± 2.6^c^
Gr_122milled_	nd	4.18 ± 0.29^b^	39.3 ± 2.5^a^	nd	nd	nd	108 ± 9.1^a^	29.2 ± 2.7^a^
Gr_210milled_	nd	8.69 ± 0.62^e,f^	41.6 ± 2.9^a^	nd	nd	nd	108 ± 8.6^a^	28.2 ± 2.1^a^

Re, non-treated red lentils; Gr, non-treated green lentils; _122_ – fermented with Lactiplantibacillus plantarum strain; _210_ – fermented with Lacticaseibacillus casei strain; _milled_ – milled lentils. TRY, tryptamine; PHE, phenylethylamine; PUT, putrescine; CAD, cadaverine; HIST, histamine; TYR, tyramine; SPRMD, spermidine; SPRM, spermine; nd, not detected. Data are represented as means (*n* = 6) ± SE. ^a–g^Means with different letters in the column are significantly different all sample groups (*p* ≤ 0.05).

Correlations between GABA and FAA with BA concentration were also analyzed in lentil samples (Table 3). Between the biogenic amine phenylethylamine (PHE) and aspartic acid (Asp), glutamic acid (Glu), glycine (Gly), methionine (Met), phenylalanine (Phe), leucine (Leu)/isoleucine (Ile) and histidine (His) moderate positive correlations were found. However, between PHE and glutamine (Gln) negative moderate correlation was established. The biogenic amine PUT showed negative correlations with serine (Ser), threonine (Thr), glycine, GABA, alanine (Ala), methionine, valine (Val), phenylalanine, leucine/isoleucine, lysine (Lys) and tyrosine (Tyr). Similar tendencies on the correlations between the biogenic amine SPRMD and FAA and GABA were found; however, SPRM showed positive correlation with aspartic acid, proline and histidine, in addition to negative correlations with threonine, glycine, GABA, methionine, valine, phenylalanine, leucine/isoleucine, lysine and tyrosine.

**Table 3:** Pearson correlations between gamma-aminobutyric acid (GABA), free amino acid (FAA) and biogenic amine (BA) concentration in lentil samples.

Amino acids	Biogenic amines							
	PHE		PUT		SPRMD		SPRM	
	*r*	*p*	*r*	*p*	*r*	*p*	*r*	*p*
Serine	−0.077	0.686	−0.549**	0.002	−0.416*	0.022	−0.353	0.055
Aspartic acid	0.472**	0.009	0.714**	0.0001	0.740**	0.0001	0.516**	0.003
Glutamic acid	0.569**	0.001	0.180	0.342	0.418*	0.021	0.225	0.233
Threonine	0.236	0.210	−0.0720**	0.0001	−0.530**	0.003	−0.608**	0.0001
Glycine	0.497**	0.005	−0.642**	0.0001	−0.487**	0.006	−0.653**	0.0001
Alanine	0.237	0.207	−0.487**	0.006	−0.282	0.131	−0.294	0.115
Proline	0.340	0.066	0.271	0.148	0.527**	0.003	0.517**	0.003
Methionine	0.424*	0.019	−0.752**	0.0001	−0.562**	0.001	−0.664**	0.0001
Valine	0.312	0.093	−0.616**	0.0001	−0.399*	0.029	−0.452*	0.012
Phenylalanine	0.463**	0.010	−0.696**	0.0001	−0.520**	0.003	−0.613**	0.0001
Leu/Ile	0.508**	0.004	−0.722**	0.0001	−0.574**	0.001	−0.685**	0.0001
Lysine	0.328	0.077	−0.794**	0.0001	−0.747**	0.0001	−0.792**	0.0001
Histidine	0.596**	0.001	0.282	0.131	0.528**	0.003	0.378*	0.040
Tyrosine	0.298	0.109	−0.0814**	0.0001	−0.774**	0.0001	−0.869**	0.0001
Glutamine	−0.461*	0.010	0.361	0.050	0.249	0.185	0.266	0.155
Arginine	−0.150	0.429	0.244	0.195	0.189	0.316	0.062	0.744
GABA	−0.132	0.486	−0.761**	0.0001	−0.704**	0.0001	−0.651**	0.0001

PHE, phenylethylamine; PUT, putrescine; SPRMD, spermidine; SPRM, spermine; GABA, gamma-aminobutyric acid; *r*, Pearson correlation; *p*, significance, correlation is significant, when *p* ≤ 0.05. ** Correlation is significant at the 0.01 level (2-tailed). * Correlation is significant at the 0.05 level (2-tailed).

The amino acid arginine can be converted to agmatine or to ornithine from which PUTR is formed during the decarboxylation pathway. The amino acid lysine can be decarboxylised into CAD. Moreover, HIS, TYR, TRYP and PHE can be formed from the amino acids histidine, tyrosine, tryptophan and phenylalanine, respectively. SPRM is formed from SPRMD, which is formed from PUTR, by SPRM synthase and SPRMD synthase, respectively (20). Physiologically, the most important BA are HIST and TYR because of their toxicity (49). These later BA were not found in lentil samples. Usually, plant-based material contain PUTR, SPRM and SPRMD and lower concentrations of HIST (21), and the European Union (EU) established legislative limit values only for HIST in fish (50). However, there are recommendations for PHE (30 mg/kg) in food (51). Indeed, it was reported about the BA present in fermented soybean products (52–56). The presence of BA has been reported in legumes, and the total BA content in lentils was established to be 130 mg/kg, with predominant CAD (57, 58). However, studies about BA content in fermented lentils are very scarce. Overall, fermented vegetables were reported as the group of products in which high quantities of PUTR (264 mg/kg) and CAD (26–35.4 mg/kg) can be formed (59). Finally, taking into consideration that the high concentration of BA (1,000 mg of total BA/kg and 8 mg of HIST) can cause serious health problems (60, 61), the control of the end-product is of foremost importance.

### 3.3. Fatty acid profile in the lentil samples

**Figure 3:**
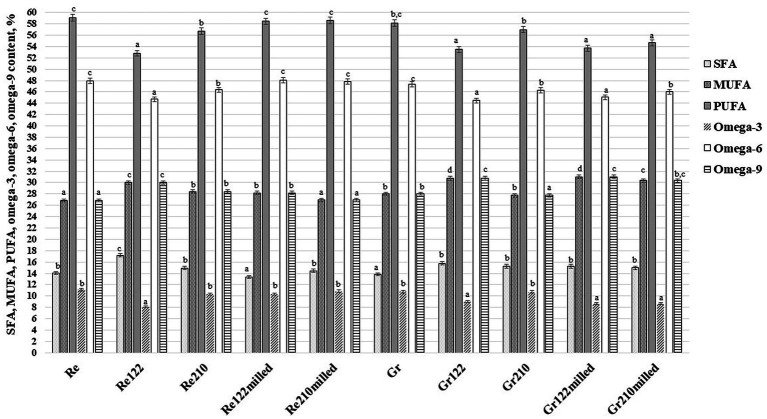
Saturated (SFA), monounsaturated (MUFA) and polyunsaturated (PUFA) fatty acid, omega-3, omega-6 and omega-9 contents in lentil samples (Re—non-treated red lentils; Gr—non-treated green lentils; _122_—fermented with *Lactiplantibacillus plantarum* strain; _210_—fermented with *Lacticaseibacillus casei* strain; _milled_—milled lentils; ^a–d^Means with different letters between the columns are significantly different, when *p* ≤ 0.05).

The FA concentrations (% from total fat content) in the lentil samples are shown in Table 4 and Figure 3. Its content in red lentil samples ranged from 44.70% (in Re_122_ samples) to, on average, 47.95% (in Re, Re_122milled_ and Re_210milled_ samples), whereas in green lentil samples ranged from, on average, 44.76% (in Gr_122_ and Gr_122milled_ samples) to, on average, 47.35% (in Gr samples). All the analyzed factors and their interactions were significant on linoleic acid content in lentil samples (Supplementary Table S1.4). Additionally, positive moderate correlation was found between pH and linoleic acid concentration in lentil samples (*r* = 0.501, *p* = 0.005), as well as moderate negative correlation was observed between LAB viable counts and linoleic acid concentration in lentil samples (*r* = −0.482, *p* = 0.007). The following dominant FA in the lentil samples was oleic acid and most of the factors and their interaction had a significant effect on this FA content (Supplementary Table S1.4). Comparing oleic acid content in red and green lentils, one found out, on average, a 3.93% higher content in green lentils. However, different tendencies of the oleic acid content in SSF samples were disclosed. Moreover, when looking among red lentil samples, the content of oleic acid in Re_122_, Re_210_ and Re_122milled_, increased but in Re_210milled_ remained similar to the control. Comparing green lentil samples, the lowest oleic acid content was found in Gr_210_ samples (on average, 27.80% from total fat content) and the highest in Gr_122milled_ samples (on average, 31.00% from total fat content). In both red and green lentil samples, SSF increased palmitic acid content. In addition, in red lentils the highest palmitic acid content was found in Re_122_ samples (on average, 15.70% from total fat content). Besides, palmitic acid content in all the fermented green lentil samples increased in comparison with the control, and the average content was 14.29%. All the analyzed factors and their interactions were significant on palmitic acid content in lentil samples but ReGr x LAB strain interaction (Supplementary Table S1.4). A negative moderate correlation between pH and palmitic acid concentration in lentil samples was unfolded (*r* = −0.573, *p* = 0.001), as well as a moderate positive correlation was found between LAB viable counts and palmitic acid (*r* = 0.606, *p* = 0.0001). In most of the cases, SSF decreased α-linolenic acid concentration in lentil samples but Gr_210_ samples. α-Linolenic acid concentration in lentils showed moderate positive correlation with pH values (*r* = 0.549, *p* = 0.002), and moderate negative correlation (*r* = −0.511, *p* = 0.004) with LAB viable counts in lentils.

**Table 4:** Fatty acid (FA) profile in lentil samples.

Lentil samples	Fatty acids				
	Palmitic acid (C16:0)	Stearic acid (C18:0)	Oleic acid (C18:1 *cis,trans*)	Linoleic acid (C18:2)	α-Linolenic acid (C18:3 α)
	Fatty acids concentration, % from total fat content				
	Parameters of the red lentil samples				
Re	12.56 ± 0.23^a^	1.50 ± 0.14^d^	26.90 ± 0.29^a^	47.97 ± 0.35^c,d^	11.12 ± 0.09^e^
Re_122_	15.70 ± 0.20^d^	1.46 ± 0.12^d^	30.00 ± 0.21^d^	44.70 ± 0.33^a^	8.13 ± 0.36^a^
Re_210_	13.52 ± 0.45^b^	1.39 ± 0.11^d^	28.40 ± 0.34^c^	46.35 ± 0.20^b^	10.33 ± 0.09^c^
Re_122milled_	13.02 ± 0.28^a,b^	0.363 ± 0.002^a^	28.20 ± 0.27^c^	48.07 ± 0.32^c,d^	10.36 ± 0.05^c^
Re_210milled_	13.57 ± 0.14^b^	0.857 ± 0.023^c^	26.90 ± 0.22^a^	47.82 ± 0.53^c,d^	10.83 ± 0.08^d^
	Parameters of the green lentil samples				
Gr	13.04 ± 0.29^a,b^	0.787 ± 0.03^6^	28.00 ± 0.16^c^	47.35 ± 0.31^c^	10.78 ± 0.07^d^
Gr_122_	14.43 ± 0.13^c^	1.31 ± 0.09^d^	30.81 ± 0.15^d^	44.47 ± 0.21^a^	9.02 ± 0.06^b^
Gr_210_	14.01 ± 0.31^c^	1.24 ± 0.08^d^	27.80 ± 0.11^b^	46.26 ± 0.22^b^	10.73 ± 0.08^d^
Gr_122milled_	14.39 ± 0.13^c^	0.858 ± 0.021^c^	31.00 ± 0.18^e^	45.05 ± 0.35^a^	8.68 ± 0.32^a,b^
Gr_210milled_	14.34 ± 0.12^c^	0.617 ± 0.014^b^	30.41 ± 0.24^d^	45.99 ± 0.13^b^	8.68 ± 0.51^a,b^

Re, non-treated red lentils; Gr, non-treated green lentils; _122_ – fermented with Lactiplantibacillus plantarum strain; _210_ – fermented with Lacticaseibacillus casei strain; _milled_ – milled lentils; SFA, saturated fatty acids, MUFA – monounsaturated fatty acids; PUFA, polyunsaturated fatty acids. Data are represented as means (*n* = 6) ± SE. ^a–e^Means with different letters in the column are significantly different all sample groups (*p* ≤ 0.05).

Saturated (SFA), monounsaturated (MUFA) and polyunsaturated (PUFA) fatty acids, and omega-3, omega-6 and omega-9 fatty acid contents in lentil samples are illustrated in Figure 3. Predominant FA groups in lentils were PUFA and MUFA, and SSF showed the tendency to increase MUFA in both red and green lentils. However, opposite trends of PUFA were obtained and their content in SSF samples was lower in comparison with the controls. Also, SSF increase SFA content in green lentils and, in most of the cases, in red lentil samples but Re_122milled_. Comparing omega-3 content in lentils, in all fermented samples, omega-3 content was lower in comparison with the controls. Similar trends were unfolded with omega-6 content: in most of the fermented samples, omega-6 content was lower but Re_122milled_. Nevertheless, opposite tendencies were disclosed regarding the omega-9 content in lentil samples, and in fermented samples in most of the cases (except Gr_210_ samples) omega-9 content was higher than the controls.

It was reported that the main FA in lentil samples are linoleic, palmitic, oleic and linolenic acids (62). Yet, in lentil samples were detected stearic, *cis* 11 eicosenoic and myristic acids. The PUFA were the major group of FA in lentils, whereas SFA were found in minor concentrations. Also, omega-6:omega-3 ratio in lentils was found to be between 1.67 and 6.65, which is in accordance with the average values described by Paucean et al. for red and green lentils (63).

The changes obtained during the fermentation can be explained by activities of the endogenous enzymes, which are present in legumes, e.g. lipoxygenase utilize unsaturated fatty acids to release volatile compounds—some of which possessing undesirable odors (64). This is especially a problem for legumes with a high proportion of unsaturated fatty acids (>80%). The evolution and presence of volatile compounds are discussed further in the next section.

### 3.4. Volatile compounds profile in the lentil samples

**Figure 4:**
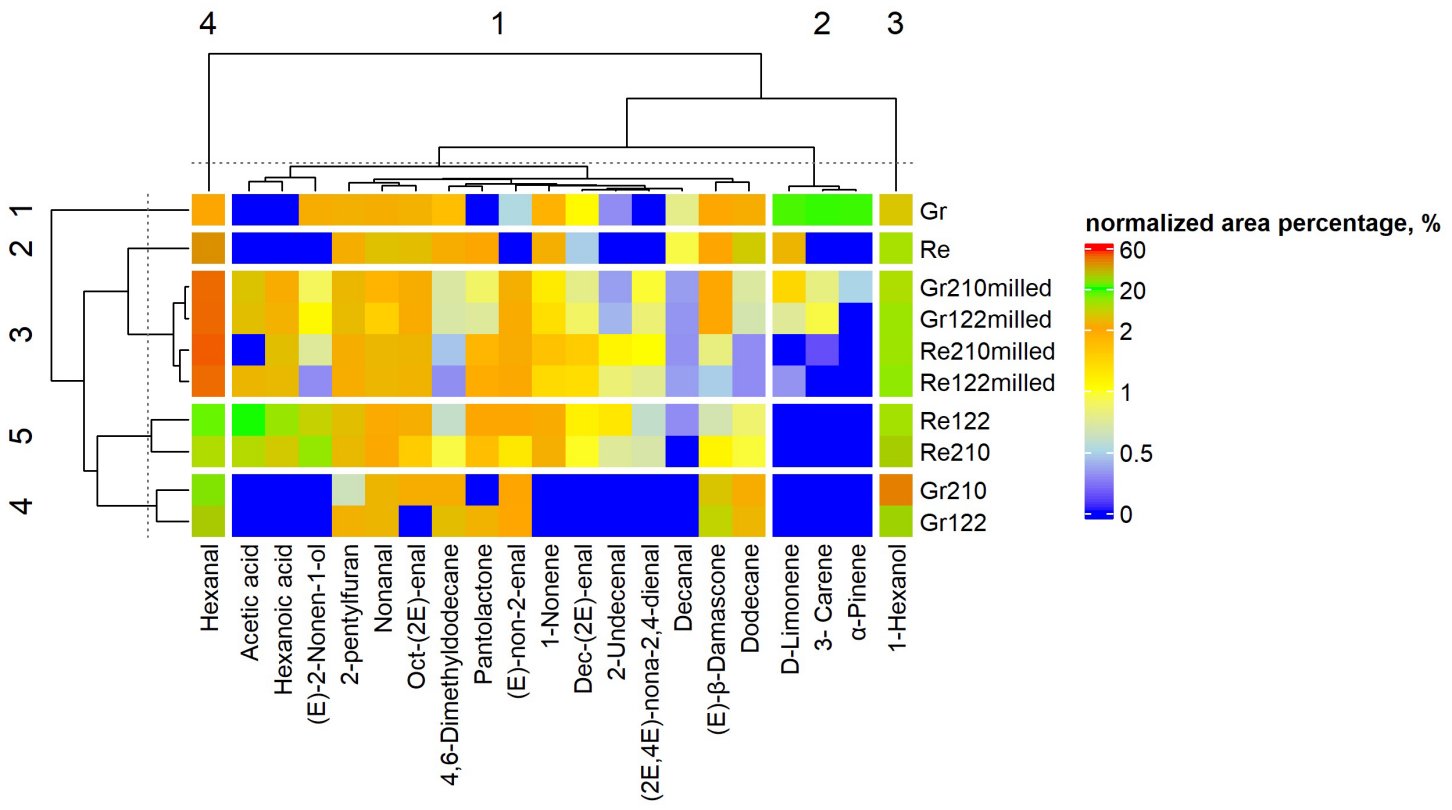
Volatile compound (VC) profile (% from the total volatile compounds content) in lentil samples (Re—non-treated red lentils; Gr—non-treated green lentils; _122_—fermented with *Lactiplantibacillus plantarum* strain; _210_—fermented with *Lacticaseibacillus casei* strain; _milled_—milled lentils).

The VC profile in lentil samples (% from the total VC content) is given in Supplementary Table S3.1 and Figure 4. The main VC in lentil samples, which content (% from the total VC content) was at least in one sample higher than 10%, were hexanal, 1-hexanol, hexanoic acid, D-limonene and (E)-2-nonen-1-ol. Hexanal flavor is described as green, fatty, leafy, vegetative, fruity and clean with a woody nuance; 1-hexanol flavor is pungent, ethereal, fusel oil, fruity and alcoholic, sweet and with a green top note; hexanoic acid is sour, fatty, sweat and cheese; D-limonene is citrus, orange, fresh and sweet and (E)-2-nonen-1-ol flavor is described as green, fatty, melon and with an oily tallow nuance (Supplementary Table S3.2). Likewise hexanoic acid in non-fermented green lentils, in non-fermented red lentil samples hexanoic acid and (E)-2-nonen-1-ol were not detected. In non-fermented red and green lentils 13 and 17 VC, respectively, were identified. However, in non-milled and milled SSF with No. 122 strain 18 and 19 VC were found, respectively, as well as in non-milled and milled SSF with No. 210 strain 17 and 18 VC were identified, respectively. Despite that in non-fermented green lentils higher variety of VC was found, in fermented samples (with both tested LAB strains) 9 VC were detected. In opposite to this trend, in milled SSF samples more than two times broader spectrum of VC was formed: 20 VC in Gr_122milled_ and 21 VC in Gr_210milled_ were identified. Analyzed factors were statistically significant on most of the VC content in lentil samples (Supplementary Table S1.5). Correspondingly, most of the VC concentrations showed significant correlations with pH and LAB viable counts of the samples (Table 4). Additionally, some of the VC showed significant correlations with FA content (Supplementary Table S4.1).

It was reported that different cultivars and color of legumes have similar characteristic volatile compound profiles (64). The undesirable odors of legumes are related to the lipoxygenase-catalyzed unsaturated fatty acid oxidation (65, 66), *viz.* volatile terpenes may be formed from degradation of carotenes by either legume lipoxygenases or hydroperoxides generated from autolytic and enzyme-catalyzed lipid oxidation (67). D-limonene is considered as potential discriminant VC in lentils (64). The latter is associated with a citrus and fresh odor (68). Beany and green odors are majorly derived from hexanal and 1-octen-3-ol (65, 66, 69). Previous studies were focused on soybean volatile compounds (65, 70–73) and the experimental data concerning lentil volatile compounds are scarce (Table 5).

**Table 5:** Pearson correlations between pH and lactic acid bacteria (LAB) viable counts with volatile compound (VC) content in lentil samples.

Volatile compounds	pH 24		LAB viable counts	
	*r*	*p*	*r*	*p*
Acetic acid	−0.446*	0.013	0.359	0.051
Hexanal	−0.322	0.083	0.352	0.057
1-Hexanol	−0.368*	0.045	0.416*	0.022
α-Pinene	0.719**	0.0001	−0.655**	0.000
Hexanoic acid	−0.499**	0.005	0.404*	0.027
2-pentylfuran	−0.190	0.315	0.169	0.372
3- Carene	0.712**	0.0001	−0.647**	0.0001
D-Limonene	0.841**	0.0001	−0.782**	0.0001
Oct-(2E)-enal	0.489**	0.006	−0.503**	0.005
1-Nonene	0.331	0.074	−0.409*	0.025
Pantolactone	−0.227	0.227	0.179	0.344
Nonanal	0.400*	0.029	−0.395*	0.031
(E)-non-2-enal	−0.788**	0.0001	0.837**	0.0001
(E)-2-Nonen-1-ol	−0.170	0.368	0.108	0.569
Dodecane	0.676**	0.0001	−0.661**	0.0001
Decanal	0.841**	0.0001	−0.839**	0.0001
(2E,4E)-nona-2,4-dienal	−0.590**	0.001	0.585**	0.001
Dec-(2E)-enal	−0.054	0.779	0.018	0.923
4,6-Dimethyldodecane	0.205	0.276	−0.152	0.424
2-Undecenal	−0.403*	0.027	0.325	0.080
(E)-β-Damascone	−0.088	0.643	0.175	0.356

LAB, lactic acid bacteria; *r*, Pearson correlation; *p*, significance, correlation is significant, when *p* ≤ 0.05. ** Correlation is significant at the 0.01 level (2-tailed). * Correlation is significant at the 0.05 level (2-tailed).

The FA composition of legumes in conjunction with their VC were studied. The study conveyed that the large number (13) of terpenes and hexanal sets up the most abundant compounds in lentils (64). In opposite to aldehydes, alcohols, ketones and hydrocarbons, which are products of FA oxidation, terpenes are naturally present in plants (67), and α- and β-pinene are the most common terpenes in legumes (64). Furthermore, nonanal and 2-hexenal were the second and third most abundant VC in legumes (64). These findings are in agreement with our current study. The difference in hexanal abundance was explained by the difference in the content of linoleic and linolenic FA, because they are the precursor for lipoxygenase-catalyzed evolution of hexanal (65). Hydroperoxide lyase isozymes degrade hydroperoxides into isomeric nonenals, including hexanal (74, 75). The latter VC can be further used by enzymes, further generating additional volatile aldehydes (76), which may help to explain the presence of other analogous aldehydes. Also, legumes contain alcohol dehydrogenases, which can catalyze the interconversion of aldehydes, alcohols and acids (77), thus possibly explaining the abundance of 1-hexanol. Other groups of VC, like ketones and hydrocarbons, are also derived from both non-enzymatic and enzymatic lipid oxidation (67), however, these VC are more typical in dry beans (78).

## 4. Conclusion

From this research effort, it can be concluded that both studied LAB strains are suitable for lentil fermentation (pH < 4.5, LAB viable counts >8.0 log_10_ CFU/g) and solid-state fermentation with these strains increases essential free amino acid content in lentils. However, some of the non-essential FAA content in fermented lentils decreased. Additionally, SSF significantly increases GABA concentration in lentils, and milling process is a significant factor on GABA synthesis in the studied fermentable substrates. Predominant biogenic amine in lentils was SPRMD. In overall, SSF reduces the total BA content in lentils (on average, 34.8% in red lentils and 39.9% in green lentils). The main fatty acids in lentils were linoleic and oleic acids, and SSF showed the trend to increase MUFA and decrease PUFA contents. The main volatile compounds in lentil samples were hexanal, 1-hexanol, hexanoic acid, D-limonene and (E)-2-nonen-1-ol, and most of the VC showed significant correlations with the pH, LAB viable counts and FA of the lentil samples. Finally, LAB strain used for SSF and milling process are statistically significant factors for most of the analyzed parameters, and, despite that in green non-milled SSF lentils, higher GABA concentration was found, for both (green and red) lentils, milling and SSF combination is recommended, because these groups of samples showed the lowest BA content, in addition to high concentrations of essential FAA and GABA.

## Data availability statement

The original contributions presented in the study are included in the article/Supplementary material, further inquiries can be directed to the corresponding author.

## Author contributions

EB: conceptualization, resources, supervision, project administration, validation, and data curation. EM, VB, and DK: methodology. EM, EZ, and VS: software and visualization. EM, EZ, DK, VS, and RR: formal analysis. EM and JR: investigation. EM, DK, VS, RR, and EZ: writing—original draft preparation. JR, VB, and EB: writing—review and editing. All authors have read and agreed to the published version of the manuscript.

## Conflict of interest

The authors declare that the research was conducted in the absence of any commercial or financial relationships that could be construed as a potential conflict of interest.

## Publisher’s note

All claims expressed in this article are solely those of the authors and do not necessarily represent those of their affiliated organizations, or those of the publisher, the editors and the reviewers. Any product that may be evaluated in this article, or claim that may be made by its manufacturer, is not guaranteed or endorsed by the publisher.

## Abbreviations

Ala: Alanine
AA: Amino acid
Arg: Arginine
Asp: Aspartic acid
BA: Biogenic amines
−b*: Blueness
CAD: Cadaverine
CFU: Colony-forming units
MRS: De Man, Rogosa and Sharpe
EU: European Union
FAME: Fatty acid methyl esters
FA: Fatty acids
FAO: Food and Agriculture Organization
FAA: Free amino acids
GABA: γ-aminobutyric acid
GC: Gas chromatographer/phy
GRAS: Generally regarded as safe
Glu: Glutamic acid
Gln: Glutamine
Gly: Glycine
Gr: Green lentils
−a*: Greenness
His: Histidine
Ile: Isoleucine
LAB: Lactic acid bacteria
*Lc. casei*: *Lacticaseibacillus casei*
*Lp. plantarum*: *Lactiplantibacillus plantarum*
Leu: Leucine
L*: Lightness
Lys: Lysine
MS: Mass spectrometer/try
Met: Methionine
MUFA: Monounsaturated fatty acids
PHE: Penylethylamine
Phe: Phenylalanine
PUFA: Polyunsaturated fatty acids
Pro: Proline
PUTR: Putrescine
Re: Red lentils
a*: Redness
SFA: Saturated fatty acids
Ser: Serine
SPME: Solid-phase microextraction
SSF: Solid-state fermentation
SPRMD: Spermidine
SPRM: Spermine
SE: Standard error
Thr;: Threonine
TRP: ryptamine
TYR: Tyramine
Tyr: Tyrosine
UV/VIS: Ultra-violet/visible
Val: Valine
VC: Volatile compounds
b*: Yellowness

## References

[1] Ganesan K Xu B. Polyphenol-rich lentils and their health promoting effects. Int J Mol Sci. (2017) 18:2390. doi: 10.3390/ijms18112390, PMID: 29125587 PMC5713359

[2] Mudgal V Mehta MK Rane AS. Lentil straw (lens Culinaris): an alternative and nutritious feed resource for kids. Anim Nutr. (2018) 4:417–21. doi: 10.1016/j.aninu.2018.04.009, PMID: 30564762 PMC6286626

[3] Faris MA-IE Takruri HR Issa AY. Role of lentils (lens *Culinaris* L.) in human health and nutrition: a review. Mediterr J Nutr Metab. (2013) 6:3–16. doi: 10.1007/s12349-012-0109-8

[4] Food and Agriculture Organization (FAO) Traditional food plants, FAO: Rome, Italy. Available at: https://www.fao.org/3/W0078E/w0078e12.htm (Accessed November 27, 2022).

[5] Xu B Chang SK. Phenolic substance characterization and chemical and cell-based antioxidant activities of 11 lentils grown in the northern United States. J Agric Food Chem. (2010) 58:1509–17. doi: 10.1021/jf903532y, PMID: 20058926

[6] FAOSTAT. Statistics. Available at: http://www.fao.org/statistics/en/ (Accessed November 27, 2022).

[7] Ryan E Galvin K O’Connor TP Maguire AR O’Brien NM. Phytosterol, Squalene, Tocopherol content and fatty acid profile of selected seeds, grains, and legumes. Plant Foods Hum Nutr. (2007) 62:85–91. doi: 10.1007/s11130-007-0046-8, PMID: 17594521

[8] Grusak MA. Nutritional and health-beneficial quality/the lentil—botany, production and uses. Wallingford Comm Agric Bur. (2009):368–90. doi: 10.1079/9781845934873.0368

[9] US Department of Agriculture FoodData central. Available at: https://fdc.nal.usda.gov/ (Accessed November 27, 2022)

[10] Dhull SB Kinabo J Uebersax MA. Nutrient profile and effect of processing methods on the composition and functional properties of lentils (lens Culinaris Medik): a review. Legum Sci. (2023) 5:e156. doi: 10.1002/leg3.156

[11] Bartkiene E Sakiene V Bartkevics V Rusko J Lele V Juodeikiene G. Lactofermentation and protein isolation: effects on phenolic compounds and Genistein, antioxidant properties, trypsin inhibitor activity, and protein digestibility. Eur Food Res Technol. (2018) 244:1521–31. doi: 10.1007/s00217-018-3066-8

[12] Xiao Y Xing G Rui X Li W Chen X Jiang M. Enhancement of the antioxidant capacity of chickpeas by solid state fermentation with Cordyceps Militaris SN-18. J Funct Foods. (2014) 10:210–22. doi: 10.1016/j.jff.2014.06.008

[13] Dey TB Chakraborty S Jain KK Sharma A Kuhad RC. Antioxidant Phenolics and their microbial production by submerged and solid state fermentation process: a review. Trends Food Sci Technol. (2016) 53:60–74.

[14] Magro AEA Silva LC Rasera GB de Castro RJS. Solid-state fermentation as an efficient strategy for the biotransformation of lentils: enhancing their antioxidant and Antidiabetic potentials. Bioresour Bioprocess. (2019) 6:1–9. doi: 10.1186/s40643-019-0273-5

[15] Dhull SB Punia S Kidwai MK Kaur M Chawla P Purewal SS. Solid-state fermentation of lentil (lens *Culinaris* L.) with Aspergillus Awamori: effect on phenolic compounds, mineral content, and their bioavailability. Legume Sci. (2020) 2:37. doi: 10.1002/leg3.37

[16] Soetan KO Oyewole OE. The need for adequate processing to reduce the anti-nutritional factors in plants used as human foods and animal feeds: a review. Afr J Food Sci. (2009) 3:223–32.

[17] Grewal J. Gamma-Aminobutyric acid (GABA): a versatile bioactive compound. Eur J Mol Clin Med. (2020) 7:3068–75.

[18] Diana M Quílez J Rafecas M. Gamma-Aminobutyric acid as a bioactive compound in foods: a review. J Funct Foods. (2014) 10:407–20. doi: 10.1016/j.jff.2014.07.004

[19] Li H Qiu T Huang G Cao Y. Production of gamma-Aminobutyric acid by lactobacillus Brevis NCL912 using fed-batch fermentation. Microb Cell Factories. (2010) 9:85. doi: 10.1186/1475-2859-9-85, PMID: 21070676 PMC2996345

[20] Sarkadi LS. Amino acids and biogenic amines as food quality factors. Pure Appl Chem. (2019) 91:289–300. doi: 10.1515/pac-2018-0709, PMID: 36668894

[21] Sarkadi LS. Biogenic amines In: Process-Induced Food Toxicants. eds. Stadler R. H. Lineback D. R. John Wiley & Sons, Ltd (2008). Hoboken, NJ, 321–361.

[22] Bartkiene E Starkute V Katuskevicius K Laukyte N Fomkinas M Vysniauskas E. The contribution of edible cricket flour to quality parameters and sensory characteristics of wheat bread. Food Sci Nutr. (2022) 10:4319–30. doi: 10.1002/fsn3.3024, PMID: 36514776 PMC9731535

[23] Ben-Gigirey B Vieites Baptista de Sousa JM Villa TG Barros-Velazquez J. Histamine and Cadaverine production by bacteria isolated from fresh and frozen albacore (Thunnus Alalunga). J Food Prot. (1999) 62:933–9. doi: 10.4315/0362-028X-62.8.933, PMID: 10456749

[24] Ben-Gigirey B Vieites Baptista de Sousa JM Villa TG Barros-Velazquez J. Changes in biogenic amines and microbiological analysis in albacore (Thunnus Alalunga) muscle during frozen storage. J Food Prot. (1998) 61:608–15. doi: 10.4315/0362-028X-61.5.608, PMID: 9709235

[25] Pérez-Palacios T Ruiz-Carrascal J Solomando JC Antequera T. Strategies for enrichment in ω-3 fatty acids aiming for healthier meat products. Food Rev Int. (2019) 35:485–503. doi: 10.1080/87559129.2019.1584817

[26] Mousavi M-H Gharekhani M Alirezalu K Roufegarinejad L Azadmard-Damirchi S. Production and characterization of nondairy gluten-free fermented beverage based on buckwheat and lentil. Food Sci Nutr. (2021) 11:1342–53. doi: 10.1002/fsn3.3170, PMID: 37181300 PMC10171538

[27] Chen K Gao C Han X Li D Wang H Lu F. Co-fermentation of lentils using lactic acid bacteria and bacillus Subtilis Natto increases functional and antioxidant components. J Food Sci. (2021) 86:475–83. doi: 10.1111/1750-3841.15349, PMID: 32964467

[28] Bartkiene E Lele V Ruzauskas M Domig KJ Starkute V Zavistanaviciute P. Lactic acid bacteria isolation from spontaneous sourdough and their characterization including antimicrobial and antifungal properties evaluation. Microorganisms. (2020) 8:64. doi: 10.3390/microorganisms8010064, PMID: 31905993 PMC7023352

[29] Papadimitriou K Pratsinis H Nebe-von-Caron G Kletsas D Tsakalidou E. Acid tolerance of streptococcus Macedonicus as assessed by flow Cytometry and single-cell sorting. Appl Environ Microbiol. (2007) 73:465–76. doi: 10.1128/AEM.01244-06, PMID: 17098924 PMC1796968

[30] Zhang B Peng H Deng Z Tsao R. Phytochemicals of lentil (lens Culinaris) and their antioxidant and anti-inflammatory effects. J Food Bioact. (2018) 1:93–103. doi: 10.31665/JFB.2018.1128

[31] Zhang B Deng Z Tang Y Chen P Liu R Ramdath DD. Fatty acid, carotenoid and Tocopherol compositions of 20 Canadian lentil cultivars and synergistic contribution to antioxidant activities. Food Chem. (2014) 161:296–304. doi: 10.1016/j.foodchem.2014.04.014, PMID: 24837953

[32] Liu QH Shao T Bai YF. The effect of Fibrolytic enzyme, lactobacillus Plantarum and two food antioxidants on the fermentation quality, alpha-Tocopherol and Beta-carotene of high moisture Napier grass silage ensiled at different temperatures. Anim Feed Sci Technol. (2016) 221:1–11. doi: 10.1016/j.anifeedsci.2016.08.020

[33] Hubert J Berger M Nepveu F Paul F Daydé J. Effects of fermentation on the phytochemical composition and antioxidant properties of soy germ. Food Chem. (2008) 109:709–21. doi: 10.1016/j.foodchem.2007.12.081, PMID: 26049983

[34] El Hag ME El Tinay AH Yousif NE. Effect of fermentation and Dehulling on starch, Total polyphenols, Phytic acid content and in vitro protein digestibility of pearl millet. Food Chem. (2002) 77:193–6. doi: 10.1016/S0308-8146(01)00336-3

[35] Nkhata SG Ayua E Kamau EH Shingiro J-B. Fermentation and germination improve nutritional value of cereals and legumes through activation of endogenous enzymes. Food Sci Nutr. (2018) 6:2446–58. doi: 10.1002/fsn3.846, PMID: 30510746 PMC6261201

[36] Pranoto Y Anggrahini S Efendi Z. Effect of natural and lactobacillus Plantarum fermentation on in-vitro protein and starch Digestibilities of sorghum flour. Food Biosci. (2013) 2:46–52. doi: 10.1016/j.fbio.2013.04.001

[37] Osman MA. Effect of traditional fermentation process on the nutrient and Antinutrient contents of pearl millet during preparation of Lohoh. J Saudi Soc Agric Sci. (2011) 10:1–6. doi: 10.1016/j.jssas.2010.06.001

[38] Bartkiene E Sakiene V Bartkevics V Juodeikiene G Lele V Wiacek C. Modulation of the nutritional value of lupine Wholemeal and protein isolates using submerged and solid-state fermentation with Pediococcus Pentosaceus strains. Int J Food Sci Technol. (2018) 53:1896–905. doi: 10.1111/ijfs.13775

[39] Sahab NRM Subroto E Balia RL Utama GL. γ-Aminobutyric acid found in fermented foods and beverages: current trends. Heliyon. (2020) 6:e05526. doi: 10.1016/j.heliyon.2020.e05526, PMID: 33251370 PMC7680766

[40] Ohmori T Tahara M Ohshima T. Mechanism of gamma-Aminobutyric acid (GABA) production by a lactic acid bacterium in yogurt-sake. Process Biochem. (2018) 74:21–7. doi: 10.1016/j.procbio.2018.08.030, PMID: 36829524

[41] Wu C-H Hsueh Y-H Kuo J-M Liu S-J. Characterization of a potential probiotic lactobacillus Brevis RK03 and efficient production of γ-Aminobutyric acid in batch fermentation. Int J Mol Sci. (2018) 19:143. doi: 10.3390/ijms19010143, PMID: 29300336 PMC5796092

[42] Hong J Kim K-J. Crystal structure of γ-Aminobutyrate aminotransferase in complex with a PLP-GABA adduct from Corynebacterium Glutamicum. Biochem Biophys Res Commun. (2019) 514:601–6. doi: 10.1016/j.bbrc.2019.04.194, PMID: 31072617

[43] Han S-M Lee J-S. Production and its anti-hyperglycemic effects of γ-Aminobutyric acid from the wild yeast strain Pichia Silvicola UL6-1 and Sporobolomyces Carnicolor 402-JB-1. Mycobiology. (2017) 45:199–203. doi: 10.5941/MYCO.2017.45.3.199, PMID: 29138625 PMC5673516

[44] Li H Cao Y. Lactic acid bacterial cell factories for gamma-Aminobutyric acid. Amino Acids. (2010) 39:1107–16. doi: 10.1007/s00726-010-0582-7, PMID: 20364279

[45] Rai AK Pandey A Sahoo D. Biotechnological potential of yeasts in functional food industry. Trends Food Sci Technol. (2019) 83:129–37. doi: 10.1016/j.tifs.2018.11.016, PMID: 36643101

[46] Kim D-H Dasagrandhi C Park S-K Eom S-H Huh M-K Mok J-S. Optimization of gamma-Aminobutyric acid production using sea tangle extract by lactic acid bacterial fermentation. Lwt. (2018) 90:636–42. doi: 10.1016/j.lwt.2018.01.011

[47] Liao W-C Wang C-Y Shyu Y-T Yu R-C Ho K-C. Influence of preprocessing methods and fermentation of adzuki beans on γ-Aminobutyric acid (GABA) accumulation by lactic acid bacteria. J Funct Foods. (2013) 5:1108–15. doi: 10.1016/j.jff.2013.03.006

[48] Kumar S Punekar NS. The metabolism of 4-Aminobutyrate (GABA) in fungi. Mycol Res. (1997) 101:403–9. doi: 10.1017/S0953756296002742

[49] Ladero V Calles-Enriquez M Fernandez M Alvarez MA. Toxicological effects of dietary biogenic amines. Curr Nutr Food Sci. (2010) 6:145–56. doi: 10.2174/157340110791233256, PMID: 36541202

[50] Directive E. Council Directive 91/493/EEC of 22 July 1991 laying down the health conditions for the production and the placing on the market of fishery products. JL. (1991) 268:0015–34.

[51] ten Brink B Damink C Joosten HMLJ Huis in ‘t veld JHJ. Occurrence and formation of biologically active amines in foods. Int J Food Microbiol. (1990) 11:73–84. doi: 10.1016/0168-1605(90)90040-C, PMID: 2223522

[52] Kung H-F Tsai Y-H Wei C-I. Histamine and other biogenic amines and histamine-forming bacteria in miso products. Food Chem. (2007) 101:351–6. doi: 10.1016/j.foodchem.2005.12.057

[53] Shukla S Park H-K Kim J-K Kim M. Determination of biogenic amines in Korean traditional fermented soybean paste (Doenjang). Food Chem Toxicol. (2010) 48:1191–5. doi: 10.1016/j.fct.2010.01.034, PMID: 20146930

[54] Tsai Y-H Chang S-C Kung H-F. Histamine contents and histamine-forming bacteria in Natto products in Taiwan. Food Control. (2007) 18:1026–30. doi: 10.1016/j.foodcont.2006.06.007

[55] Yongmei L Xiaohong C Mei J Xin L Rahman N Mingsheng D. Biogenic amines in Chinese soy sauce. Food Control. (2009) 20:593–7. doi: 10.1016/j.foodcont.2008.08.020, PMID: 35730767

[56] Guan R-F Liu Z-F Zhang J-J Wei Y-X Wahab S Liu D-H. Investigation of biogenic amines in Sufu (Furu): a Chinese traditional fermented soybean food product. Food Control. (2013) 31:345–52. doi: 10.1016/j.foodcont.2012.10.033

[57] Simon-Sarkadi L Holzapfel W-H. Biogenic amines and microbial quality of sprouts. Z. Für Lebensm.-Unters. Forsch. (1995) 200:261–5. doi: 10.1007/BF01187516 7785356

[58] Skowronek F Simon-Sarkadi L Holzapfel WH. Hygienic status and biogenic amine content of Mung bean sprouts. Zeitschrift für Untersuchung der Lebensmittel-Untersuchung und -Forschung. (1998) 207:97–100. doi: 10.1007/s002170050301

[59] EFSA Scientific opinion on risk based control of biogenic amine formation in fermented foods | EFSA. Available at: https://www.efsa.europa.eu/en/efsajournal/pub/2393 (Accessed July 26, 2022)

[60] Sivamaruthi BS Kesika P Chaiyasut C. A narrative review on biogenic amines in fermented fish and meat products. J Food Sci Technol. (2021) 58:1623–39. doi: 10.1007/s13197-020-04686-x, PMID: 33897002 PMC8021659

[61] Erdag D Merhan O Yildiz B. Biochemical and pharmacological properties of biogenic amines. Biog Amines. (2018) 8:1–14. doi: 10.5772/intechopen.81569

[62] Lastras C Revilla I González-Martín MI Vivar-Quintana AM. Prediction of fatty acid and mineral composition of lentils using near infrared spectroscopy. J Food Compos Anal. (2021) 102:104023. doi: 10.1016/j.jfca.2021.104023

[63] Socaci SA Dulf FV Alexa E Man SMM An AE Muste S. Folic acid, minerals, amino-acids, fatty acids and volatile compounds of green and red lentils. Folic acid content optimization in wheat-lentils composite flours. Chem Cent J. (2018) 12:88. doi: 10.1186/s13065-018-0456-8, PMID: 30078060 PMC6078380

[64] Khrisanapant P Kebede B Leong SY Oey I. A comprehensive characterisation of volatile and fatty acid profiles of legume seeds. Foods. (2019) 8:651. doi: 10.3390/foods8120651, PMID: 31817745 PMC6963610

[65] MacLeod G Ames J Betz NL. Soy flavor and its improvement. Crit Rev Food Sci Nutr. (1988) 27:219–400. doi: 10.1080/10408398809527487, PMID: 3067980

[66] Rackis JJ Sessa DJ Honig DH. Flavor Problems of Vegetable Food Proteins. J Am Oil Chem Soc. (1979) 56:262–71. doi: 10.1007/BF02671470, PMID: 36890873

[67] Azarnia S Boye JI. Flavour Compounds in Legumes: Chemical and Sensory Aspects. New York: Prog. Food Sci. Technol. Nova Science Publishers (2011).

[68] Mosciano G Pohero W Michalski J Holmgren C Young D. Organoleptic characteristics of flavor materials. Perfum Flavorist. (2000) 25:71–4. doi: 10.1097/00006205-200025020-00005

[69] Roland WS Pouvreau L Curran J van de Velde F de Kok PM. Flavor aspects of pulse ingredients. Cereal Chem. (2017) 94:58–65. doi: 10.1094/CCHEM-06-16-0161-FI

[70] Zhang Y Guo S Liu Z Chang SK. Off-flavor related volatiles in soymilk as affected by soybean variety, grinding, and heat-processing methods. J Agric Food Chem. (2012) 60:7457–62. doi: 10.1021/jf3016199, PMID: 22812487

[71] Li Y-Q Zhang W-N. Effects of pulsed electric fields on volatile constituents of soymilk. Agro Food Ind Hi-Tech. (2012) 23:36–7.

[72] Achouri A Boye JI Zamani Y. Soybean variety and storage effects on soymilk flavour and quality. Int J Food Sci Technol. (2008) 43:82–90. doi: 10.1111/j.1365-2621.2006.01393.x, PMID: 30333634

[73] Min S Yu Y Yoo S Martin SS. Effect of soybean varieties and growing locations on the flavor of soymilk. J Food Sci. (2005) 70:C1–C11. doi: 10.1111/j.1365-2621.2005.tb09009.x

[74] Olias JM Rios JJ Valle M Zamora R Sanz LC Axelrod B. Fatty acid Hydroperoxide Lyase in germinating soybean seedlings. J Agric Food Chem. (1990) 38:624–30. doi: 10.1021/jf00093a009

[75] Matoba T Hidaka H Kitamura K Kaizuma N Kito M. Contribution of Hydroperoxide Lyase activity to N-Hexanal formation in soybean. J Agric Food Chem. (1985) 33:856–8. doi: 10.1021/jf00065a022

[76] Liu K. Soybeans: Chemistry, Technology, and Utilization Springer (2012). Berlin p.

[77] Gomes J Jadrić S Winterhalter M Brkić S. Alcohol dehydrogenase Isoenzymes in chickpea cotyledons. Phytochemistry. (1982) 21:1219–24. doi: 10.1016/0031-9422(82)80114-3

[78] Oomah BD Liang LS Balasubramanian P. Volatile compounds of dry beans (Phaseolus Vulgaris L.). Plant Foods Hum Nutr. (2007) 62:177–83. doi: 10.1007/s11130-007-0059-3, PMID: 17926127

