# Supplementary material for: Influence of different lactic acid bacteria strains and milling process on the solid-state fermented green and red lentils (Lens culinaris L.) properties including gamma-aminobutyric acid formation
Source: Front Nutr. 2023 Apr 13;10:1118710. doi: 10.3389/fnut.2023.1118710 (PMC10133501; doi:10.3389/fnut.2023.1118710)
Supplement: Supplementary file 1 [file Table_1.DOCX]

**Table S1.1.** Significance (p) of the analysed factors and their interactions on pH, color coordinates and lactic acid bacteria (LAB) viable counts in lentil samples.

| **Dependent Variable** | **Analysed factors and their interactions** | | | | | | |
| --- | --- | --- | --- | --- | --- | --- | --- |
|  | Re or Gr | LAB strain used for fermentation | Milled or non-milled | ReGr * LAB strain used for fermentation | ReGr * Milled or non-milled | LAB strain used for fermentation * Milled or non-milled | ReGr * LAB strain used for fermentation * Milled or non-milled |
|  | Significance (p) of the analysed factors and their interactions on analysed lentil parameters | | | | | | |
| pH after 24 h | 0.0001 | 0.0001 | 0.785 | 0.0001 | 0.0001 | 0.022 | 0.022 |
| L* | 0.080 | 0.0001 | 0.0001 | 0.0001 | 0.0001 | 0.548 | 0.791 |
| a* | 0.0001 | 0.0001 | 0.0001 | 0.0001 | 0.183 | 0.718 | 0.323 |
| b* | 0.0001 | 0.0001 | 0.0001 | 0.0001 | 0.513 | 0.009 | 0.003 |
| LAB count | 0.021 | 0.0001 | 0.384 | 0.436 | 0.0001 | 0.936 | 0.873 |
| L* – lightness; a* – redness (−a* greenness); b* – yellowness (−b* blueness); LAB – lactic acid bacteria; Re –red lentils; Gr –green lentils; Factor or factors interaction is significant, when p ≤ 0.05 | | | | | | | |

**Table S1.2**. Significance (p) of the analysed factors and their interactions on gamma-aminobutyric acid (GABA) and free amino acid (FAA) content in lentil samples.

| **Dependent Variable** | **Analysed factors and their interactions** | | | | | | |
| --- | --- | --- | --- | --- | --- | --- | --- |
|  | Re or Gr | LAB strain used for fermentation | Milled or non-milled | ReGr * LAB strain used for fermentation | ReGr * Milled or non-milled | LAB strain used for fermentation * Milled or non-milled | ReGr * LAB strain used for fermentation * Milled or non-milled |
|  | Significance (p) of the analysed factors and their interactions on analysed lentil parameters | | | | | | |
| Ser | 0.0001 | 0.0001 | 0.0001 | 0.0001 | 0.0001 | 0.148 | 0.0001 |
| Asp | 0.033 | 0.0001 | 0.0001 | 0.002 | 0.375 | 0.297 | 0.653 |
| Glu | 0.0001 | 0.0001 | 0.0001 | 0.0001 | 0.0001 | 0.069 | 0.020 |
| Gly | 0.0001 | 0.0001 | 0.0001 | 0.025 | 0.015 | 0.088 | 0.0001 |
| Ala | 0.005 | 0.825 | 0.0001 | 0.012 | 0.890 | 0.169 | 0.935 |
| Pro | 0.360 | 0.0001 | 0.057 | 0.0001 | 0.0001 | 0.173 | 0.384 |
| Tyr | 0.0001 | 0.0001 | 0.0001 | 0.0001 | 0.0001 | 0.0001 | 0.242 |
| Gln | 0.0001 | 0.005 | 0.327 | 0.691 | 0.936 | 0.022 | 0.056 |
| Arg | 0.0001 | 0.0001 | 0.0001 | 0.047 | 0.0001 | 0.035 | 0.025 |
| Thr | 0.0001 | 0.0001 | 0.0001 | 0.031 | 0.304 | 0.041 | 0.001 |
| Met | 0.0001 | 0.0001 | 0.0001 | 0.0001 | 0.023 | 0.0001 | 0.050 |
| Val | 0.0001 | 0.0001 | 0.0001 | 0.0001 | 0.828 | 0.0001 | 0.035 |
| Phe | 0.032 | 0.0001 | 0.0001 | 0.071 | 0.284 | 0.0001 | 0.003 |
| Leu/Ile | 0.0001 | 0.002 | 0.014 | 0.012 | 0.005 | 0.166 | 0.069 |
| Lys | 0.0001 | 0.0001 | 0.0001 | 0.009 | 0.102 | 0.005 | 0.0001 |
| His | 0.0001 | 0.021 | 0.0001 | 0.0001 | 0.0001 | 0.0001 | 0.002 |
| GABA | 0.001 | 0.0001 | 0.028 | 0.0001 | 0.0001 | 0.0001 | 0.0001 |
| Val – valine; Leu – leucine; Ile – isoleucine; Thr – threonine; Met – methionine; Phe – phenylalanine; Lys – lysine; His – histidine; GABA - gamma-aminobutyric acid; Ala – alanine; Gly – glycine; Ser – serine; Pro – proline; Asp – aspartic acid; Glu – glutamic acid; Gln – glutamine; Tyr – tyrosine; Arg - arginine. LAB – lactic acid bacteria; Re –red lentils; Gr –green lentils; Factor or factors interaction is significant, when p ≤ 0.05 | | | | | | | |

**Table S1.3**. Significance (p) of the analysed factors and their interactions on biogenic amine (BA) concentration in lentil samples.

| **Dependent Variable** | **Analysed factors and their interactions** | | | | | | |
| --- | --- | --- | --- | --- | --- | --- | --- |
|  | Re or Gr | LAB strain used for fermentation | Milled or non-milled | ReGr * LAB strain used for fermentation | ReGr * Milled or non-milled | LAB strain used for fermentation * Milled or non-milled | ReGr * LAB strain used for fermentation * Milled or non-milled |
|  | Significance (p) of the analysed factors and their interactions on analysed lentil parameters | | | | | | |
| PHE | 0.0001 | 0.0001 | 0.0001 | 0.0001 | 0.0001 | 0.0001 | 0.0001 |
| PUT | 0.072 | 0.0001 | 0.0001 | 0.301 | 0.063 | 0.977 | 0.664 |
| SPRMD | 0.0001 | 0.0001 | 0.0001 | 0.966 | 0.878 | 0.509 | 0.576 |
| SPRM | 0.0001 | 0.0001 | 0.0001 | 0.857 | 0.044 | 0.364 | 0.795 |
| PHE – phenylethylamine; PUT – putrescine; SPRMD – spermidine; SPRM – spermine; LAB – lactic acid bacteria; Re –red lentils; Gr –green lentils; Factor or factors interaction is significant, when p ≤ 0.05 | | | | | | | |

**Table S1.4**. Significance (p) of the analysed factors and their interactions on fatty acid (FA) concentration in lentil samples.

| **Dependent Variable** | **Analysed factors and their interactions** | | | | | | |
| --- | --- | --- | --- | --- | --- | --- | --- |
|  | Re or Gr | LAB strain used for fermentation | Milled or non-milled | ReGr * LAB strain used for fermentation | ReGr * Milled or non-milled | LAB strain used for fermentation * Milled or non-milled | ReGr * LAB strain used for fermentation * Milled or non-milled |
|  | Significance (p) of the analysed factors and their interactions on analysed lentil parameters | | | | | | |
| Palmitic acid | 0.008 | 0.0001 | 0.005 | 0.071 | 0.001 | 0.0001 | 0.005 |
| Stearic acid | 0.0001 | 0.0001 | 0.0001 | 0.0001 | 0.0001 | 0.004 | 0.0001 |
| Oleic acid | 0.0001 | 0.0001 | 0.176 | 0.0001 | 0.0001 | 0.0001 | 0.0001 |
| Linoleic acid | 0.0001 | 0.0001 | 0.0001 | 0.023 | 0.0001 | 0.0001 | 0.050 |
| α-Linolenic acid | 0.0001 | 0.0001 | 0.377 | 0.0001 | 0.0001 | 0.0001 | 0.958 |
| SFA | 0.342 | 0.001 | 0.002 | 0.897 | 0.026 | 0.025 | 0.045 |
| MUFA | 0.069 | 0.114 | 0.915 | 0.882 | 0.137 | 0.506 | 0.615 |
| PUFA | 0.266 | 0.209 | 0.494 | 0.955 | 0.236 | 0.441 | 0.890 |
| Omega-3 | 0.010 | 0.0001 | 0.779 | 0.319 | 0.0001 | 0.009 | 0.987 |
| Omega-6 | 0.341 | 0.382 | 0.390 | 0.967 | 0.448 | 0.644 | 0.860 |
| Omega-9 | 0.082 | 0.137 | 0.919 | 0.892 | 0.155 | 0.526 | 0.631 |
| SFA – saturated fatty acids, MUFA – monounsaturated fatty acids, PUFA – polyunsaturated fatty acids.  LAB – lactic acid bacteria; Re –red lentils; Gr –green lentils; Factor or factors interaction is significant, when p ≤ 0.05. | | | | | | | |

**Table S1.5**. Significance (p) of the analysed factors and their interactions on volatile compound (VC) profile in lentil samples.

| **Dependent Variable** | **Analysed factors and their interactions** | | | | | | |
| --- | --- | --- | --- | --- | --- | --- | --- |
|  | Re or Gr | LAB strain used for fermentation | Milled or non-milled | ReGr * LAB strain used for fermentation | ReGr * Milled or non-milled | LAB strain used for fermentation * Milled or non-milled | ReGr * LAB strain used for fermentation * Milled or non-milled |
|  | Significance (p) of the analysed factors and their interactions on analysed lentil parameters | | | | | | |
| Acetic acid | 0.0001 | 0.0001 | 0.0001 | 0.0001 | 0.0001 | 0.001 | 0.015 |
| Hexanal | 0.0001 | 0.206 | 0.0001 | 0.0001 | 0.0001 | 0.033 | 0.766 |
| 1-Hexanol | 0.003 | 0.0001 | 0.0001 | 0.0001 | 0.0001 | 0.0001 | 0.726 |
| α-Pinene | 0.0001 | 0.0001 | 0.686 | 0.0001 | 0.686 | 0.686 | 0.686 |
| Hexanoic acid | 0.0001 | 0.0001 | 0.004 | 0.0001 | 0.0001 | 0.0001 | 0.0001 |
| 2-pentylfuran | 0.617 | 0.0001 | 0.206 | 0.0001 | 0.0001 | 0.0001 | 0.067 |
| 3- Carene | 0.0001 | 0.0001 | 0.028 | 0.0001 | 0.056 | 0.997 | 0.740 |
| D-Limonene | 0.0001 | 0.0001 | 0.033 | 0.0001 | 0.103 | 0.717 | 0.357 |
| Oct-(2E)-enal | 0.0001 | 0.0001 | 0.0001 | 0.0001 | 0.047 | 0.173 | 0.0001 |
| 1-Nonene | 0.0001 | 0.0001 | 0.040 | 0.0001 | 0.0001 | 0.291 | 0.704 |
| Pantolactone | 0.0001 | 0.0001 | 0.0001 | 0.0001 | 0.0001 | 0.0001 | 0.0001 |
| Nonanal | 0.0001 | 0.0001 | 0.011 | 0.0001 | 0.0001 | 0.398 | 0.979 |
| (E)-non-2-enal | 0.0001 | 0.0001 | 0.0001 | 0.001 | 0.371 | 0.0001 | 0.061 |
| (E)-2-Nonen-1-ol | 0.0001 | 0.0001 | 0.0001 | 0.0001 | 0.0001 | 0.001 | 0.002 |
| Dodecane | 0.014 | 0.0001 | 0.0001 | 0.0001 | 0.0001 | 0.004 | 0.001 |
| Decanal | 0.0001 | 0.0001 | 0.0001 | 0.0001 | 0.001 | 0.001 | 0.004 |
| (2E,4E)-nona-2,4-dienal | 0.0001 | 0.0001 | 0.0001 | 0.0001 | 0.0001 | 0.009 | 0.851 |
| Dec-(2E)-enal | 0.0001 | 0.001 | 0.0001 | 0.0001 | 0.0001 | 0.125 | 0.013 |
| 4,6-Dimethyldodecane | 0.0001 | 0.0001 | 0.0001 | 0.0001 | 0.0001 | 0.0001 | 0.0001 |
| 2-Undecenal | 0.0001 | 0.0001 | 0.0001 | 0.0001 | 0.0001 | 0.0001 | 0.0001 |
| (E)-β-Damascone | 0.0001 | 0.0001 | 0.0001 | 0.0001 | 0.0001 | 0.001 | 0.0001 |
| LAB – lactic acid bacteria; Re –red lentils; Gr –green lentils; Factor or factors interaction is significant, when p ≤ 0.05 | | | | | | | |
