# Supplementary material for: Influence of different lactic acid bacteria strains and milling process on the solid-state fermented green and red lentils (Lens culinaris L.) properties including gamma-aminobutyric acid formation
Source: Front Nutr. 2023 Apr 13;10:1118710. doi: 10.3389/fnut.2023.1118710 (PMC10133501; doi:10.3389/fnut.2023.1118710)
Supplement: Supplementary file 2 [file Table_2.DOCX]

**Table S2.1.** Free amino acid (FAA) and gamma-aminobutyric acid (GABA) content in lentil samples.

| **Lentil**  **samples** | **Essential free amino acid (FAA) and GABA content, µmol/g** | | | | | | | | |
| --- | --- | --- | --- | --- | --- | --- | --- | --- | --- |
|  | **Thr** | **Met** | **Val** | **Phe** | **Leu/Ile** | **Lys** | **His** | **GABA** | |
|  | Parameters of the red lentil samples | | | | | | | | |
| Re | 0.937  ±0.034b | 0.042  ±0.006b | 1.11  ±0.13b | 0.240  ±0.021a | 0.449  ±0.037a | 0.407  ±0.056a | 1.05  ±0.15d | nd | |
| Re_122_ | 1.81  ±0.21d | 0.266  ±0.039d | 2.14  ±0.28e | 0.731  ±0.082c | 2.22  ±0.17d | 1.08  ±0.16d | 0.773  ±0.067b | 4.53  ±0.35c | |
| Re_210_ | 1.12  ±0.12c | 0.151  ±0.02c | 1.08  ±0.15b | 0.389  ±0.055b | 1.09  ±0.16c | 0.780  ±0.087c | 0.714  ±0.057b | 2.91  ±0.29b | |
| Re_122milled_ | 3.12  ±0.40d | 0.542  ±0.056f | 2.46  ±0.18e | 1.01  ±0.07d | 4.23  ±0.28e | 1.59  ±0.14e | 1.01  ±0.15d | 8.46  ±0.64e | |
| Re_210milled_ | 3.10  ±0.27d | 0.578  ±0.043f | 2.29  ±0.28e | 1.08  ±0.10d | 4.30  ±0.37e | 1.60  ±0.16e | 0.915  ±0.032c | 8.13  ±0.76e | |
|  | Parameters of the green lentil samples | | | | | | | | |
| Gr | 0.444  ±0.053a | 0.029  ±0.004a | 0.718  ±0.063a | 0.273  ±0.028a | 0.397  ±0.048a | 0.587  ±0.085b | 0.718  ±0.055b | 0.174  ±0.016a | |
| Gr_122_ | 1.33  ±0.17c | 0.112  ±0.016c | 1.16  ±0.17b | 0.346  ±0.028b | 0.827  ±0.067b | 1.34  ±0.12d | 0.672  ±0.065b | 9.35  ± 0.81e | |
| Gr_210_ | 1.10  ±0.15c | 0.140  ±0.017c | 1.08  ±0.11b | 0.397  ±0.059b | 0.752  ±0.052b | 0.919  ±0.083d | 0.624  ±0.053a,b | 8.48  ±0.59e | |
| Gr_122milled_ | 3.13  ±0.469e | 0.332  ±0.046d,e | 1.90  ±0.15d | 0.608  ±0.086c | 2.28  ±0.29d | 1.29  ±0.15d | 0.528  ±0.056a | 5.77  ±0.47d | |
| Gr_210milled_ | 1.49  ±0.21c | 0.355  ±0.028e | 1.57  ±0.13c | 0.837  ±0.084c | 4.43  ±0.49e | 2.02  ±0.23f | 0.730  ±0.023b | 5.19  ±0.29d | |
|  | Nonessential free amino acid (FAA) content, µmol/g | | | | | | | | |
|  | **Ser** | **Asp** | **Glu** | **Gly** | **Ala** | **Pro** | **Tyr** | **Gln** | **Arg** |
|  | Parameters of the red lentil samples | | | | | | | | |
| Re | 0.927  ±0.082a | 6.52  ±0.53f | 9.80  ±0.84f | 0.950  ±0.135b | 2.44  ±0.23b | 5.06  ±0.44d | 0.064  ±0.005a | 5.27  ±0.49d | 8.52  ±0.79f |
| Re_122_ | 2.96  ±0.23e | 1.48  ±0.12c | 3.45  ±0.29c,d | 1.22  ±0.10b | 6.60  ±0.56d | 5.16  ±0.52d | 0.212  ±0.021b | 3.64  ±0.32b | 2.73  ±0.21a |
| Re_210_ | 1.68  ±0.17c | 0.750  ±0.085b | 2.86  ±0.26a | 1.36  ±0.12b | 7.26  ±0.59e | 3.97  ±0.31c | 0.192  ±0.014b | 2.91  ±0.22a | 3.79  ±0.35b |
| Re_122milled_ | 2.40  ±0.22d | 3.27  ±0.26e | 7.94  ±0.58e | 2.64  ±0.21c | 8.95  ±0.43f | 4.26  ±0.34c,d | 0.741  ±0.054f | 4.13  ±0.35b,c | 5.16  ±0.41c |
| Re_210milled_ | 2.01  ±0.27c,d | 1.97  ±0.28d | 7.07  ±0.62e | 2.54  ±0.32c | 6.31  ±0.51d | 4.56  ±0.32d | 0.648  ±0.046e,f | 3.13  ±0.20a,b | 7.26  ±0.32e |
|  | Parameters of the green lentil samples | | | | | | | | |
| Gr | 0.843  ±0.124a | 7.47  ±0.61f | 3.75  ±0.40c,d | 1.21  ±0.12b | 1.83  ±0.23a | 2.25  ±0.34b | 0.159  ±0.019b | 6.09  ±0.55d | 7.71  ±0.53e,f |
| Gr_122_ | 1.13  ±0.13b | 0.446  ±0.052a | 3.62  ±0.32c | 0.820  ±0.028a | 1.44  ±0.12a | 2.38  ±0.21b | 0.439  ±0.031d | 5.24  ±0.46d | 5.86  ±0.51d |
| Gr_210_ | 1.40  ±0.13b,c | 0.486  ±0.044a | 2.85  ±0.32a | 0.998  ±0.052b | 1.66  ±0.12a | 1.67  ±0.14a | 0.348  ±0.025c | 9.39  ±0.85e | 9.77  ±0.54g |
| Gr_122milled_ | 2.71  ±0.23e | 1.72  ±0.24c,d | 3.17  ±0.26b,c | 2.20  ±0.20c | 6.67  ±0.57d | 2.12  ±0.21b | 0.633  ±0.051e | 3.22  ±0.21b | 7.40  ±0.57e,f |
| Gr_210milled_ | 1.61  ±0.24c | 1.53  ±0.11c | 4.44  ±0.46d | 2.17  ±0.27c | 4.81  ±0.42c | 1.57  ±0.13a | 0.953  ±0.087g | 3.55  ±0.25b | 5.16  ±0.46c,d |
| Val – valine; Leu – leucine; Ile – isoleucine; Thr – threonine; Met – methionine; Phe – phenylalanine; Lys – lysine; His – histidine; GABA - gamma-aminobutyric acid; Ala – alanine; Gly – glycine; Ser – serine; Pro – proline; Asp – asparagine; Glu – glutamic acid; Gln – glutamine; Tyr – tyrosine; Arg - arginine. Data are represented as means (n = 6) ± SE. nd – not detected; ^a-g^ Means with different letters in the lines are significantly different all sample groups (p ≤ 0.05). | | | | | | | | | |
