# Supplementary material for: Influence of different lactic acid bacteria strains and milling process on the solid-state fermented green and red lentils (Lens culinaris L.) properties including gamma-aminobutyric acid formation
Source: Front Nutr. 2023 Apr 13;10:1118710. doi: 10.3389/fnut.2023.1118710 (PMC10133501; doi:10.3389/fnut.2023.1118710)
Supplement: Supplementary file 3 [file Table_3.DOCX]

Table S3.1. Volatile compound (VC) profile (% from the total volatile compounds content) in lentil samples.

| **Volatile compounds**  (% from the total volatile compound content) | **Lentil samples** | | | | | | | | | |
| --- | --- | --- | --- | --- | --- | --- | --- | --- | --- | --- |
|  | Re | Re_122_ | Re_210_ | Re_122milled_ | Re_210milled_ | Gr | Gr_122_ | Gr_210_ | Gr_122milled_ | Gr_210milled_ |
| Acetic acid | nd | 20.2  ±1.65d | 11.6  ±1.27c | 4.81  ±0.48a | nd | nd | nd | nd | 6.39  ±0.34b | 7.09  ±0.64b |
| Hexanal | 45.4  ±4.4f | 17.5  ±1.09c | 11.9  ±1.46b | 51.7  ±3.58g | 53.8  ±4.26g | 2.15  ±0.21a | 33.0  ±3.12e | 26.8  ±3.03d | 52.2  ±5.60g | 51.9  ±5.08g |
| 1-Hexanol | 12.8  ±0.97b | 13.0  ±1.00b | 32.4  ±3.17c | 14.8  ±1.60b | 13.8  ±1.77b | 7.43  ±0.84a | 31.0  ±1.57c | 48.5  ±5.34d | 13.9  ±1.01b | 12.3  ±1.27b |
| α-Pinene | nd | nd | nd | nd | nd | 21.3  ±2.31b | nd | nd | nd | 0.491  ±0.055a |
| Hexanoic acid | nd | 14.1  ±1.8d | 8.21  ±0.90c | 5.32  ±0.45b | 6.30  ±0.61b | nd | nd | nd | 3.86  ±0.33a | 3.11  ±0.32a |
| 2-pentylfuran | 3.46  ±0.36b | 6.36  ±0.82d | 5.16  ±0.30c | 3.18  ±0.16b | 3.47  ±0.32b | 3.72  ±0.30b | 3.93  ±0.41b | 0.633  ±0.061a | 5.41  ±0.46c | 4.94  ±0.56c |
| 3- Carene | nd | nd | nd | nd | 0.131  ±0.012a | 21.0  ±1.52c | nd | nd | 0.934  ±0.120b | 0.800  ±0.095b |
| D-Limonene | 4.66  ±0.41d | nd | nd | 0.315  ±0.021a | nd | 22.0  ±2.07e | nd | nd | 0.735  ±0.053b | 1.45  ±0.12c |
| Oct-(2E)-enal | 6.12  ±0.64d | 3.43  ±0.22b | 1.56  ±0.18a | 4.32  ±0.46c | 4.63  ±0.27c | 3.84  ±0.49b,c | nd | 3.28  ±0.21b | 2.96  ±0.32b | 3.17  ±0.24b |
| 1-Nonene | 3.58  ±0.45d | 2.98  ±0.36c | 3.52  ±0.31d | 1.41  ±0.11a | 1.68  ±0.09b | 1.85  ±0.13b | nd | nd | 1.36  ±0.11a | 1.23  ±0.11a |
| Pantolactone | 2.16  ±0.18b | 2.13  ±0.25b | 1.72  ±0.15b | 1.92  ±0.20b | 1.82  ±0.20b | nd | 4.14  ±0.45c | nd | 0.726  ±0.064a | 0.870  ±0.099a |
| Nonanal | 6.65  ±0.82f | 2.71  ±0.19c | 2.65  ±0.29c | 4.71  ±0.54e | 4.96  ±0.28e | 3.49  ±0.23d | 4.82  ±0.57e | 4.82  ±0.50e | 1.54  ±0.09a | 1.83  ±0.16b |
| (E)-non-2-enal | nd | 2.15  ±0.20c | 1.26  ±0.12b | 2.67  ±0.13d | 3.00  ±0.30e | 0.515  ±0.031a | 1.98  ±0.21c | 2.01  ±0.16c | 2.93  ±0.17e | 3.60  ±0.23f |
| (E)-2-Nonen-1-ol | nd | 9.81  ±1.15e | 14.5  ±1.83f | 0.294  ±0.022a | 0.731  ±0.093b | 3.14  ±0.22d | nd | nd | 1.06  ±0.09c | 0.889  ±0.084b |
| Dodecane | 8.44  ±0.66f | 0.851  ±0.081c | 0.954  ±0.069c | 0.295  ±0.015a | 0.294  ±0.021a | 3.27  ±0.42d | 4.71  ±0.30e | 3.15  ±0.23d | 0.670  ±0.083b | 0.713  ±0.043b |
| Decanal | 0.935  ±0.099c | 0.296  ±0.036a | nd | 0.347  ±0.019a | 0.309  ±0.017a | 0.776  ±0.082b | nd | nd | 0.318  ±0.028a | 0.340  ±0.030a |
| (2E,4E)-nona-2,4-dienal | nd | 0.583  ±0.055a | 0.688  ±0.086a,b | 0.758  ±0.038b | 1.01  ±0.11c | nd | nd | nd | 0.828  ±0.068b | 0.962  ±0.082b,c |
| Dec-(2E)-enal | 0.471  ±0.029a | 1.15  ±0.14c,d | 0.981  ±0.067c | 1.38  ±0.15d | 1.57  ±0.19d | 1.36  ±0.08d | nd | nd | 0.872  ±0.087b,c | 0.778  ±0.044b |
| 4,6-Dimethyldodecane | 3.17  ±0.20g | 0.590  ±0.047c | 0.936  ±0.056e | 0.301  ±0.035a | 0.448  ±0.038b | 1.72  ±0.10f | 6.00  ±0.67h | 3.19  ±0.34g | 0.695  ±0.050d | 0.707  ±0.045d |
| 2-Undecenal | nd | 1.27  ±0.13d | 0.732  ±0.079c | 0.827  ±0.061c | 1.11  ±0.05d | 0.294  ±0.035a | nd | nd | 0.406  ±0.039b | 0.345  ±0.026a,b |
| (E)-β-Damascone | 2.00  ±0.13e | 0.664  ±0.077b | 1.10  ±0.09d | 0.472  ±0.047a | 0.808  ±0.051c | 2.37  ±0.29e | 10.2  ±1.16g | 7.50  ±0.62f | 2.23  ±0.13e | 2.38  ±0.17e |
| VC – volatile compound; nd – not detected; Data are represented as means (n = 6) ± SE. ^a-g^ Means with different letters between the columns are significantly different all sample groups (p ≤ 0.05). | | | | | | | | | | |

Table S3.2. Flavour description of the separate volatile compounds (VC).

| **Volatile compounds** | ***Flavour description** |
| --- | --- |
| Acetic acid | pungent sour overripe fruit |
| Hexanal | Green, fatty, leafy, vegetative, fruity and clean with a woody nuance |
| 1-Hexanol | Pungent, etherial, fusel oil, fruity and alcoholic, sweet with a green top note |
| α-Pinene | fresh camphor sweet pine earthy woody |
| Hexanoic acid | sour fatty sweat cheese |
| 2-pentylfuran | Fruity, green, earthy beany with vegetable like nuances |
| 3- Carene | Sweet, diffusive, terpenic |
| D-Limonene | citrus orange fresh sweet |
| Oct-(2E)-enal | fresh cucumber fatty green herbal banana waxy green leaf |
| 1-Nonene | Odor of gasoline |
| Pantolactone | - |
| Nonanal | waxy aldehydic rose fresh orris orange peel fatty peely |
| (E)-non-2-enal | Green, cucumber, aldehydic, fatty with a citrus nuance |
| (E)-2-Nonen-1-ol | Green, fatty, melon, with an oily tallow nuance |
| Dodecane | Gasoline-like to odorless |
| Decanal | sweet waxy orange peel citrus floral fatty floral citrus orange-peel |
| (2E,4E)-nona-2,4-dienal | fatty melon waxy green violet leaf cucumber tropical fruit chicken fat |
| Dec-(2E)-enal | Waxy, fatty, earthy, coriander, green, mushroom, aldehydic with a chicken and pork fat nuance |
| 4,6-Dimethyldodecane | - |
| 2-Undecenal | fresh fruity orange peel |
| (E)-β-Damascone | fruity floral berry plum blackcurrant honey rose tobacco |
| *****From TGSC Information System | |
