# Supplementary material for: Influence of different lactic acid bacteria strains and milling process on the solid-state fermented green and red lentils (Lens culinaris L.) properties including gamma-aminobutyric acid formation
Source: Front Nutr. 2023 Apr 13;10:1118710. doi: 10.3389/fnut.2023.1118710 (PMC10133501; doi:10.3389/fnut.2023.1118710)
Supplement: Supplementary file 4 [file Table_4.DOCX]

Table S4.1. Correlations between volatile compounds (VA) and fatty acids (FA) in lentil samples.

| **Volatile compounds** | r and p | **Fatty acids** | | | | | | | | | | |
| --- | --- | --- | --- | --- | --- | --- | --- | --- | --- | --- | --- | --- |
|  |  | Palmitic acid (C16:0) | Stearic acid (C18:0) | Oleic acid (C18:1 *cis,trans*) | Linoleic acid (C18:2) | α-Linolenic acid (C18:3 α) | SFA | MUFA | PUFA | Omega-3 | Omega-6 | Omega-9 |
| Acetic acid | r | 0.611** | 0.226 | 0.425* | -0.448* | -0.622** | 0.544** | 0.289 | -0.224 | -0.538** | -0.131 | 0.285 |
|  | p | 0.0001 | 0.231 | 0.019 | 0.013 | 0.0001 | 0.002 | 0.121 | 0.235 | 0.002 | 0.489 | 0.127 |
| Hexanal | r | -0.049 | -0.408* | 0.081 | 0.213 | -0.063 | -0.120 | 0.145 | 0.143 | -0.010 | 0.201 | 0.145 |
|  | p | 0.797 | 0.025 | 0.672 | 0.259 | 0.740 | 0.528 | 0.444 | 0.449 | 0.960 | 0.288 | 0.445 |
| 1-Hexanol | r | 0.140 | 0.422* | -0.023 | -0.245 | 0.166 | 0.281 | 0.064 | 0.056 | 0.189 | -0.003 | 0.076 |
|  | p | 0.460 | 0.020 | 0.905 | 0.192 | 0.382 | 0.132 | 0.738 | 0.768 | 0.318 | 0.988 | 0.692 |
| α-Pinene | r | -0.277 | -0.228 | -0.176 | 0.242 | 0.278 | -0.275 | -0.077 | 0.153 | 0.260 | 0.118 | -0.069 |
|  | p | 0.138 | 0.226 | 0.353 | 0.198 | 0.137 | 0.141 | 0.686 | 0.419 | 0.165 | 0.536 | 0.716 |
| Hexanoic acid | r | 0.510** | 0.141 | 0.167 | -0.211 | -0.415* | 0.448* | 0.155 | -0.088 | -0.346 | -0.010 | 0.152 |
|  | p | 0.004 | 0.458 | 0.378 | 0.263 | 0.023 | 0.013 | 0.413 | 0.645 | 0.061 | 0.957 | 0.421 |
| 2-Pentylfuran | r | 0.491** | 0.120 | 0.583** | -0.408* | -0.636** | 0.455* | 0.499** | -0.117 | -0.501** | 0.001 | 0.489** |
|  | p | 0.006 | 0.527 | 0.001 | 0.025 | 0.0001 | 0.012 | 0.005 | 0.539 | 0.005 | 0.994 | 0.006 |
| 3- Carene | r | -0.271 | -0.244 | -0.154 | 0.228 | 0.260 | -0.276 | -0.069 | 0.141 | 0.240 | 0.108 | -0.064 |
|  | p | 0.147 | 0.194 | 0.417 | 0.226 | 0.166 | 0.141 | 0.715 | 0.459 | 0.201 | 0.570 | 0.738 |
| D-Limonene | r | -0.365* | -0.173 | -0.240 | 0.320 | 0.339 | -0.326 | -0.115 | 0.193 | 0.315 | 0.154 | -.107 |
|  | p | 0.048 | 0.362 | 0.201 | 0.084 | 0.067 | 0.078 | 0.546 | 0.306 | 0.090 | 0.416 | 0.575 |
| Oct-(2E)-enal | r | -0.381* | -0.160 | -0.637** | 0.754** | 0.495** | -0.317 | -0.284 | 0.444* | 0.490** | 0.428* | -0.269 |
|  | p | 0.038 | 0.398 | 0.0001 | 0.0001 | 0.005 | 0.088 | 0.128 | 0.014 | 0.006 | 0.018 | 0.151 |
| 1-Nonene | r | -0.141 | 0.378* | -0.307 | 0.301 | 0.161 | 0.016 | -0.110 | 0.220 | 0.178 | 0.229 | -0.101 |
|  | p | 0.457 | 0.039 | 0.099 | 0.106 | 0.394 | 0.934 | 0.562 | 0.242 | 0.348 | 0.224 | 0.596 |
| Pantolactone | r | 0.173 | 0.356 | 0.228 | -0.231 | -0.215 | 0.263 | 0.219 | -0.024 | -0.142 | -0.002 | 0.209 |
|  | p | 0.360 | 0.054 | 0.225 | 0.219 | 0.253 | 0.160 | 0.244 | 0.902 | 0.453 | 0.990 | 0.268 |
| Nonanal | r | -0.429* | 0.266 | -0.643** | 0.557** | 0.679** | -0.207 | -0.255 | 0.469** | 0.679** | 0.377* | -0.243 |
|  | p | 0.018 | 0.156 | 0.0001 | 0.001 | 0.0001 | 0.273 | 0.174 | 0.009 | 0.0001 | 0.040 | 0.195 |
| (E)-non-2-enal | r | 0.483** | -0.526** | 0.484** | -0.265 | -.510** | 0.247 | 0.376* | -0.115 | -0.409* | -0.011 | 0.370* |
|  | p | 0.007 | 0.003 | 0.007 | 0.157 | .004 | 0.188 | 0.040 | 0.544 | 0.025 | 0.953 | 0.044 |
| (E)-2-Nonen-1-ol | r | 0.263 | 0.430* | 0.070 | -0.224 | -.164 | 0.343 | 0.076 | -0.043 | -0.129 | -0.027 | 0.084 |
|  | p | 0. 160 | 0.018 | 0.712 | 0.235 | .386 | 0.064 | 0.691 | 0.821 | 0.497 | 0.889 | 0.660 |
| Dodecane | r | -0.343 | 0.540** | -0.280 | 0.162 | .381* | -0.090 | -0.124 | 0.182 | 0.363* | 0.115 | -0.118 |
|  | p | 0.064 | 0.002 | 0.133 | 0.394 | .038 | 0.636 | 0.513 | 0.336 | 0.049 | 0.545 | 0.534 |
| Decanal | r | -0.436* | -0.102 | -0.372* | 0.555** | .339 | -0.358 | -0.162 | 0.299 | 0.329 | 0.296 | -0.154 |
|  | p | 0.016 | 0.591 | 0.043 | 0.001 | .067 | 0.052 | 0.393 | 0.108 | 0.076 | 0.113 | 0.416 |
| (2E,4E)-nona-2,4-dienal | r | 0.225 | -0.477** | 0.205 | 0.046 | -.314 | 0.052 | 0.179 | 0.0001 | -0.250 | 0.103 | 0.182 |
|  | p | 0.231 | 0.008 | 0.278 | 0.809 | .091 | 0.786 | 0.343 | 0.999 | 0.183 | 0.589 | 0.336 |
| Dec-(2E)-enal | r | -0.068 | -0.464** | -0.211 | 0.437* | .064 | -0.164 | -0.036 | 0.236 | 0.102 | 0.285 | -0.035 |
|  | p | 0.723 | 0.010 | 0.263 | 0.016 | .735 | 0.386 | 0.848 | 0.209 | 0.593 | 0.127 | 0.854 |
| 4,6-Dimethyldodecane | r | -0.009 | 0.494** | 0.108 | -0.276 | .089 | 0.170 | 0.126 | -0.002 | 0.114 | -0.061 | 0.121 |
|  | p | 0.962 | 0.006 | 0.569 | 0.139 | .639 | 0.370 | 0.508 | 0.992 | 0.549 | 0.749 | 0.523 |
| 2-Undecenal | r | 0.306 | -0.144 | -0.047 | 0.075 | -.197 | 0.207 | 0.026 | 0.041 | -0.151 | 0.100 | 0.028 |
|  | p | 0.100 | 0.447 | 0.805 | 0.692 | .297 | 0.273 | 0.892 | 0.829 | 0.424 | 0.599 | 0.885 |
| (E)-β-Damascone | r | 0.186 | 0.305 | 0.292 | -0.447* | -.071 | 0.262 | 0.235 | -0.092 | -0.029 | -0.134 | 0.229 |
|  | p | 0.326 | 0.101 | 0.118 | 0.013 | .709 | 0.161 | 0.211 | 0.630 | 0.878 | 0.481 | 0.223 |
| r – Pearson correlation; p – significance; SFA – saturated fatty acids, MUFA – monounsaturated fatty acids, PUFA – polyunsaturated fatty acids.  ** Correlation is significant at the 0.01 level (2-tailed). * Correlation is significant at the 0.05 level (2-tailed). | | | | | | | | | | | | |
